# Peripheral blood cellular immunophenotype in depression: a systematic review and meta-analysis

**DOI:** 10.1038/s41380-022-01919-7

**Published:** 2022-12-28

**Authors:** Éimear M. Foley, Joel T. Parkinson, Ruth E. Mitchell, Lorinda Turner, Golam M. Khandaker

**Affiliations:** 1 MRC Integrative Epidemiology Unit, Population Health Sciences, Bristol Medical School, University of Bristol, Bristol, UK; 2 Centre for Academic Mental Health, Population Health Sciences, Bristol Medical School, University of Bristol, Bristol, UK; 3 Institute of Infection, Immunity and Inflammation, University of Glasgow, Glasgow, UK; 4 Cambridge Institute of Therapeutic Immunology and Infectious Disease, Department of Medicine, University of Cambridge, Cambridge, UK; 5 bit.bio, Babraham Research Campus, Cambridge, UK; 6 NIHR Bristol Biomedical Research Centre, Bristol, UK; 7 Avon and Wiltshire Mental Health Partnership NHS Trust, Bristol, UK

**Keywords:** Cell biology, Diagnostic markers, Depression

## Abstract

**Introduction:**

Meta-analyses implicate immune dysfunction in depression confirming increased levels of circulating immune proteins (e.g., cytokines) in depression cases compared to controls. White blood cells (WBC) both produce and are influenced by cytokines, and play key roles in orchestrating innate and adaptive immune responses, but their role in depression remains unclear. Therefore, a systematic review of studies of various WBC subsets in depression is required for a greater understanding of the nature of immune dysfunction in this illness.

**Methods:**

We searched PubMed and PsycINFO databases (inception to 5^th^ April 2022) and conducted a systematic review and meta-analysis of identified studies comparing absolute count and/or relative percentage of flow cytometry-derived WBC subsets between depression cases and controls. Selected studies were quality assessed. Random-effect meta-analysis was performed.

**Results:**

Thirty-three studies were included and 27 studies (*n* = 2277) were meta-analysed. We report an increase in mean absolute counts of WBC (seven studies; standardised mean difference [SMD] = 1.07; 95% CI, 0.61–1.53; *P* < 0.01; *I*^2^ = 64%), granulocytes (two studies; SMD = 2.07; 95% CI, 1.45–2.68; *P* < 0.01; *I*^2^ = 0%), neutrophils (four studies; SMD = 0.91; 95% CI, 0.23–1.58; *P* < 0.01; *I*^2^ = 82%), monocytes (seven studies; SMD = 0.60; 95% CI, 0.19–1.01; *P* < 0.01; *I*^2^ = 66%), CD4^+^ helper T cells (11 studies; SMD = 0.30; 95% CI, 0.15–0.45; *P* < 0.01; *I*^2^ = 0%), natural killer cells (11 studies; SMD = 1.23; 95% CI, 0.38–2.08; *P* < 0.01; *I*^2^ = 95%), B cells (10 studies; SMD = 0.30; 95% CI, 0.03–0.57; *P* = 0.03; *I*^2^ = 56%), and activated T cells (eight studies; SMD = 0.45; 95% CI, 0.24–0.66; *P* < 0.01; *I*^2^ = 0%) in depression, compared to controls. Fewer studies reported relative percentage, indicating increased neutrophils and decreased total lymphocytes, Th1, and Th2 cells in depression.

**Conclusions:**

Depression is characterised by widespread alterations in circulating myeloid and lymphoid cells, consistent with dysfunction in both innate and adaptive immunity. Immune cells could be useful biomarkers for illness subtyping and patient stratification in future immunotherapy trials of depression, along with cytokines, other biomarkers, and clinical measures.

## Introduction

Several lines of evidence implicate immune dysfunction in the pathogenesis of depression. Meta-analyses of case-control studies confirm elevated levels of proinflammatory cytokines, such as interleukin-6 (IL-6), and acute-phase proteins, like C-reactive protein (CRP) in blood and cerebrospinal fluid (CSF) of individuals with depression compared to controls [1–5]. Approximately one quarter of depressed patients show evidence of low-grade inflammation (defined as CRP > 3 mg/L) [6]. Higher IL-6 level in childhood is associated with increased depression risk subsequently in adulthood, in a dose-dependent manner [7]. Mendelian randomization (MR) studies that use genetic variants to address the issues of reverse causation and residual confounding suggest that IL-6 and other immune proteins may play a causal role in depression [8–11], though null findings have also been reported [12, 13]. Meta-analyses of randomised controlled trials (RCTs) have reported an antidepressant effect of anti-inflammatory drugs [14–16], further supporting a role of inflammation in depression.

While the majority of immune biomarker research to date has focused on cytokines and other immune proteins, emerging literature has begun to explore cellular immune markers in the context of depression. These studies are important for a greater understanding of the nature of immune dysfunction in depression, because circulating immune cells: (i) play a critical role in orchestrating immune response; (ii) both produce and are induced by cytokines and other immune proteins; and (iii) are established therapeutic targets in various diseases. Depression has been reported to be associated with leukocytosis, monocytosis, increased neutrophil-to-lymphocyte ratio, and increased CD4^+^/CD8^+^ T cell ratio [17–19]. An inflammation-related subgroup of depression, defined using peripheral immune cell counts, was reported to be characterised by increased neutrophil, CD4^+^ helper T cell, and intermediate monocyte counts, higher IL-6 and CRP levels, and increased illness severity [20].

While existing studies indicate alterations in circulating immune cells in depression and potential usefulness of cellular immune markers for dissecting illness heterogeneity, to our knowledge there are currently no systematic reviews and meta-analyses of peripheral blood cellular immunophenotype in depression. A comprehensive summary of this literature is required for a greater understanding of the role immune dysfunction plays in depression. Such work may help clarify the nature and extent of immune dysfunction, identify potential cellular source of cytokine alterations, provide potential immunological treatment targets, and inform the identification of inflammation-related depression subgroups for patient stratification in future immunotherapy trials.

We report a systematic review and meta-analysis of the peripheral blood cellular immunophenotype in depression. Our aim is to provide a comprehensive summary and assessment of the existing literature regarding alterations in various circulating white blood cell (WBC) subsets in depression, compared to controls, derived from flow cytometry, a state-of-the-art immunophenotyping technology.

## Methods

### Search strategy and study selection

This study was conducted in line with the Preferred Reporting Items for Systematic Reviews and Meta-Analyses (PRISMA) guidelines [21]. The PRISMA checklist is presented in Supplementary Table 1. Systematic search of the PubMed and PsycINFO databases was carried out to identify all published studies of peripheral blood cellular immunophenotype in depression from database inception to 5^th^ April 2022. Search terms incorporated key indexing terms and wildcards to maximise return and included: (depression OR depressive disorder OR major depressive disorder) AND (leukocyte count OR flow cytometry OR mass cytometry OR immunophenotype OR immunophenotyping OR peripheral blood mononuclear cell). The full search strategy is presented (Supplementary Materials). Our search was restricted to English language articles and human participants. The electronic search was complemented by hand-searching meta-analyses and review articles. All titles and abstracts were examined and relevant studies were retrieved. EMF and JTP applied the inclusion and exclusion criteria independently (detailed below) and selected the final studies for this review. Any differences were resolved by further discussion and consensus involving GMK.

Included studies were: (i) original articles; (ii) written in English; (iii) based on human subjects; (iv) cohort or case-control in design; (v) compared immune phenotype between current depression cases and controls; (vi) defined depression using clinical diagnosis, diagnostic interview using the International Classification of Diseases or the Diagnostic and Statistical Manual of Mental Disorders criteria, or using a validated depression scale score cut-off; (vii) defined controls as individuals with no lifetime history of psychiatric illness; (viii) used flow cytometry, mass cytometry, or related techniques to assess detailed peripheral white blood cell phenotype. Studies that did not primarily examine or report cellular phenotype; focused on specific groups alone (e.g., specific disease group [such as individuals with human immunodeficiency virus infection], elderly, adolescents); or other studies comprised of participants with comorbid medical illness likely to compromise interpretation of immunological data were excluded. As a result, only studies reporting the cellular phenotype of non-immunocompromised adult individuals were included.

### Data extraction and quality assessment

Data extraction was performed by EMF and JTP. The main outcome measure was absolute cell count and/or relative percentage in depressed cases and controls. The following data were also extracted for each included study: (i) authors; (ii) year of publication; (iii) country of origin; (iv) study design and setting; (v) sampling method and sample size; (vi) participant age and sex; (vii) case definition; (viii) control definition; (ix) immunophenotyping method; (x) cell types; (xi) covariates. In the case of missing data or uncertainty, authors of respective studies were contacted to provide further information.

The methodological quality of each study was assessed using an adapted version of the Newcastle-Ottawa Scale (NOS) for case-control studies [22]. One NOS scoring item (non-response rate) was deemed irrelevant for the current investigation and so, the maximum possible NOS score here was eight. EMF and JTP performed quality assessment independently. Any differences were resolved by further discussion and consensus involving GMK.

### Data synthesis and meta-analysis

Separate meta-analyses were conducted for studies reporting absolute counts and relative percentage of immune cells in depressed cases, compared to controls. However, relative percentages are considered to be a more holistic representation of the immune landscape at the time of sampling, given that relative counts are affected by changes in the parent cell type. Therefore, we focused on results for total cell counts as primary, in line with standard practice in immunology. Studies were grouped according to flow cytometry markers assessed (Supplementary Table 2). Units were harmonised across studies (Supplementary Table 3). When units were not reported, authors were contacted for this information. If no response was obtained, units were deduced based on available information and following expert opinion (REM, LT, GMK). Study results were pooled using the inverse variance method, meaning that studies with larger sample sizes were given greater weight. The standard mean difference (SMD), 95% confidence intervals (CI), and pooled prevalence of each cell type were calculated using quantitative random-effect meta-analysis and visualised using forest plots. Random-effect meta-analysis was chosen, as opposed to fixed effect, due to the evident heterogeneity between studies in setting, sampling method, and assessments. The *I*^2^ statistic was used to assess heterogeneity between studies, which describes the percentage of variability in effect estimates that is due to heterogeneity [23]. Publication bias was assessed by visual inspection of funnel plots and Egger’s test for funnel plot asymmetry. Sensitivity analyses were conducted excluding any extreme outlier(s) from primary analyses based on the report of extreme effect size (as compared to other studies included in that particular meta-analysis) and impact on heterogeneity in meta-analysis. Meta-analysis was conducted on all cell types with sufficient data (i.e., reported in ≥2 studies) using Review Manager version 5.4 [24] and R version 4.0.3 using the meta-package [25, 26].

## Results

**Fig. 1:**
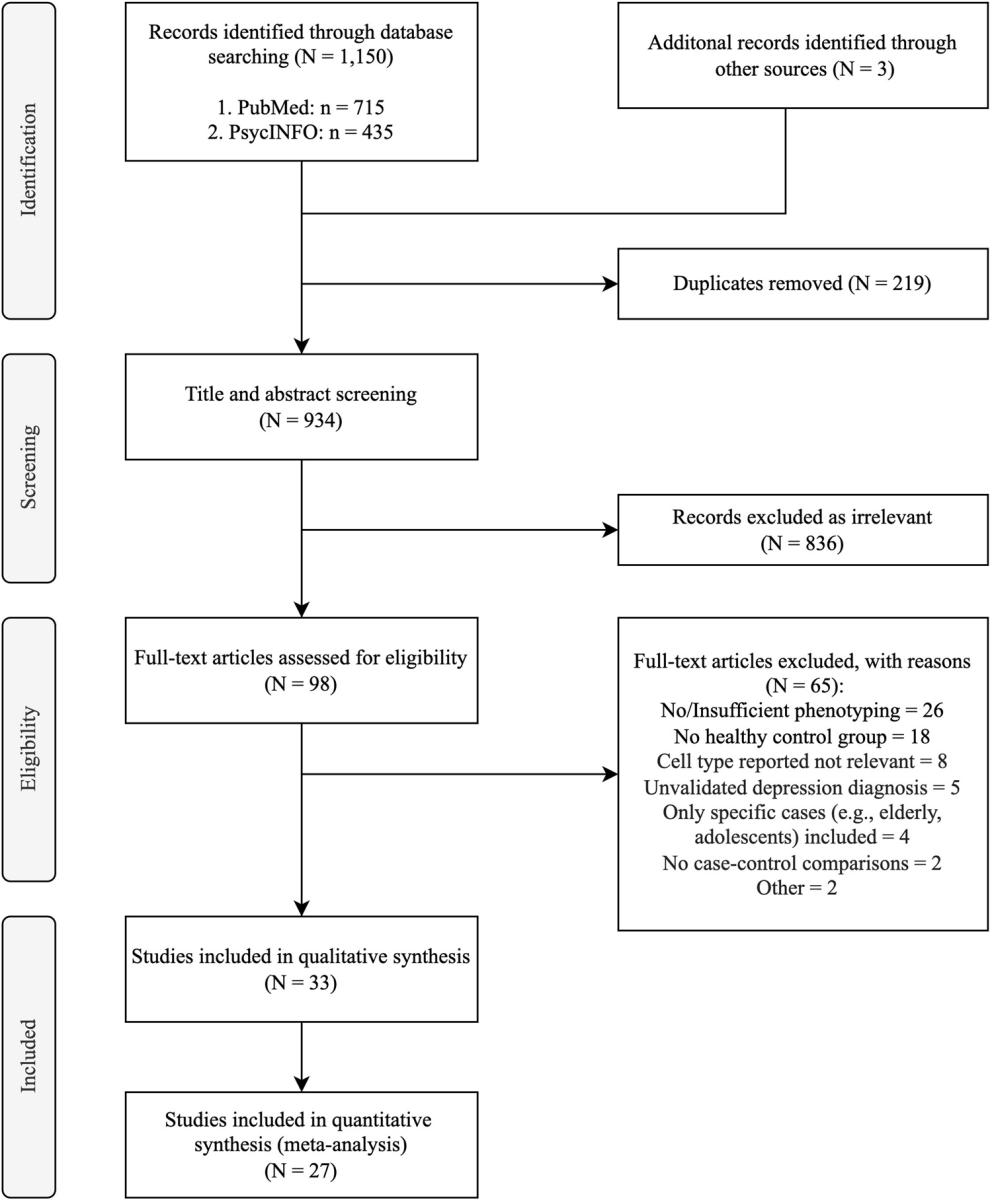
PRISMA diagram for study selection. Total number of studies identified, screened, deemed eligible, and ultimately included is summarised.

The literature search yielded 1153 potentially relevant studies. After removing duplicates, 934 studies remained. Following title and abstract screening, 98 studies were identified, of which 33 met the inclusion criteria and were included in the review [20, 27–58]. Based on data availability, meta-analysis was conducted on 27 studies (see Fig. 1 for PRISMA diagram of study selection). Table 1 presents the characteristics of included studies.

**Table 1:** Characteristics of studies included in systematic review.

No.	Study ID, country	Group (sample size)	Sex (% female)	Age in years (mean ± SD)	Sampling source	Case definition	Control definition	Immune cells assessed	Covariates	NOS Quality score^h^
1	Atanackovic, Serke & Deter (2004), Germany	Minor depression (*n* = 10) Anxiety (*n* = 13) Controls (*n* = 11)^b^	Minor depression: 80% Anxiety: 38.5% Controls: 63.6%	Depressed: 33.0 ± 7.4 Anxiety: 31.5 ± 8.6 Controls: 28.0 ± 4.2	Cases: Hospital inpatients Controls: Staff members	ICD-10 diagnostic criteria for respective disorder. Recently diagnosed infectious disease, surgical treatment, immunomodulatory or psychotropic medication, regular smokers, drinkers, or drug use excluded.	Not reported/defined.	Leukocytes, lymphocytes, neutrophils, eosinophils, basophils, CD3^+^, CD3^+^/CD4^+^, CD3^+^/CD8^+^, CD4^+^/CD8^+^ ratio, CD19^+^, CD8^+^/CD38^+^, CD3/HLA-DR^+^, CD4^+^/CD45RA^+^, CD8^+^/CD45RO^+^	None	5
2	Başterzi et al. (2010), Turkey	Depression (*n* = 69) Controls (*n* = 36)^b^	Depression: 71% Controls: 53%	Depression: 31 ± 12 Controls: 29 ± 4	Cases: Hospital inpatients Controls: Not specified	DSM-IV diagnosis of MDD or MDD recurrent. No axis I or II comorbidities. No immunomodulatory illness. No acute physical illness in the past 6 months, not pregnant, no antidepressants, non-steroidal anti-inflammatory drugs, or oral contraceptives in the last 4 weeks.	No lifetime or current diagnosis of any psychiatric disorders No immunomodulatory illness. No acute physical illness in the past 6 months, not pregnant, no antidepressants, non-steroidal anti-inflammatory drugs, or oral contraceptives in the last 4 weeks.	CD3, CD4, CD8, CD4/CD8, CD16/CD56, CD19^+^, CD45, Anti-HLA-DR	None	5
3	Becking et al. (2018), the Netherlands, Germany, Belgium	Moderate depression (*n* = 34) Severe depression (*n* = 56) Bipolar depression (*n* = 91) Controls (*n* = 165)	Moderate depression: 58.5% Severe depression: 53.6% Bipolar depression: 49% Controls: 59%	Moderate depression mean (CI): 43.6 (29.8 – 39.3) Severe depression mean (CI): 44.6 (40.9 – 48.4) Controls mean (CI): 40.3 (38.1 – 42.5)	Cases: Hospital inpatients Controls: Community	DSM-IV criteria for MDD. No inflammation-related symptoms, illnesses, or medications. No pregnancy.	No severe medical illnesses and no psychiatric illness. No inflammation-related symptoms, illnesses, or medications. No pregnancy.	Monocytes, lymphocytes, NK cells, B cells, T cells, T cytotoxic cells, T helper cells, Th17, Th2, Th1, Treg cells	Age, sex	7
4	Euteneuer et al. (2014), Germany	Depression (*n* = 38) Somatization syndromes (SSI; *n* = 25) Controls (*n* = 47)	Depression: 58% SSI: 76% Controls: 66%	Depression: 32.53 ± 12.27 SSI: 39.38 ± 14.26 Controls: 36.13 ± 13.24	Cases: Outpatient clinics Controls: Community	DSM-IV diagnosis of MDD or somatization disorder. No organic diseases of the central nervous system or affecting immune status, psychotic symptoms, alcohol/drug abuse, hormone medication (apart from contraceptive pill), stimulants, anxiolytics, current psychotherapy, pregnancy, or lactation in women	No medical or psychiatric disorders. No alcohol or drug abuse, hormone medication (apart from contraceptive pill), stimulants, anxiolytics, current psychotherapy, pregnancy, or lactation in women.	CD3^+^, CD19^+^, CD3^−^/CD56^+^, CD3^+^CD4^+^, CD3^+^CD8^+^, CD3^+^CD45RO^+^, CD3^+^CD25^+^, CD3^+^HLADR^+^, CD3^+^CD45RA^+^, CD14^+^	Age, sex, physical activity, BMI, physical activity variation	7
5	Ghosh et al. (2020), India	Depression (*n* = 53) Controls (*n* = 53)	Depression: 40% Controls: 36%	Depression: 29.2 ± 7.8 Controls: 30.9 ± 7.8	Cases: Hospital outpatients Controls: Community	ICD-10 classification of MDD. No comorbid psychiatric disorders, medical illnesses, or history of immunotherapy. Select medications excluded. No pregnant or lactating females.	No psychiatric disease, medical illnesses or immunomodulating medications. No pregnant or lactating females.	T_h_17, T_reg_ cells, CD4^+^, T_h_17/T_reg_ ratio	Age	6
6	Grosse et al. (2016a), the Netherlands	Depression (*n* = 40) Controls (*n* = 40)^b^	Depression: 60% Controls: 50%	Depression: 52 ± 9 Controls: 49 ± 9	Cases: Hospital inpatients Controls: Community	DSM-IV-TR diagnosis of MDD with melancholic features and ≥17 on the HAMD. Select other psychiatric disorders excluded. No alcohol of drug dependence or serious medical condition. No infections or allergic reactions at least 2 weeks prior. No pregnancy or breastfeeding.	No medical or psychiatric disorders. No infections or allergic reactions for at least 2 weeks prior.	Monocytes, CD19^+^, CD3^−^CD56^+^, CD3^+^, CD3^+^CD8^+^, CD3^+^CD4^+^, T_h_1, T_h_2, T_h_17, T_reg_ cells	Age, gender	7
7	Grosse et al. (2016b), Germany	Depression (*n* = 71) Controls (*n* = 71)^b^	Depression: 62% Controls: 66%	Depression: 33 ± 12 Controls: 31 ± 11	Cases: Hospital inpatients Controls: Community	DSM-IV diagnosis of MDD with no comorbid psychiatric disorder or immunomodulatory illness.	No history of psychiatric illness, or immunomodulatory illness.	CD14^+^, lymphocytes, CD19^+^, CD3^−^CD56^+^, CD3^+^, CD3^+^CD4^−^CD8^+^, CD3^+^CD4^+^CD8^−^, T_h_1, T_h_2, T_h_17, T_reg_ cells	Age, gender, BMI, smoking	7
8	Hasselmann et al. (2018), Germany	Depression (*n* = 35) Controls (*n* = 35)^f^	Depression: 71% Controls: 71%	Depression: 31.7 ± 11.2 Control: 31.7 ± 10.2	Cases: Hospital inpatients, referrals, community Controls: Community	Clinician confirmed diagnosis of MDD. No comorbid psychiatric illness other than mild-moderate anxiety. No major medical illness or immunomodulatory treatment. No anti-depressant medication 2 weeks prior.	No major medical or psychiatric disorders. No affective disorder in first-degree relative. No immunomodulatory medication. MADRS score <7.	Lymphocytes, monocytes, CD4^+^, CD8^+^, B cells, NK cells, Treg cells, CD3^−^, CD20, CD56, HLA-DR	None	7
9	Hernandez et al. (2010), Mexico	Depression (*n* = 31) Controls (*n* = 22)^c^	Depression: 77% Controls: 68%	Depression: 32 ± 9 Controls: 31 ± 6	Cases: Outpatient Clinics Controls: Not specified	DSM-IV diagnosis of MDD. No major medical illness. Not pregnant, low caffeine, alcohol and tobacco consumption.	No medical or psychiatric disorders. Not receiving medication for any illness, not pregnant, low caffeine, alcohol and tobacco consumption.	NK cells, B cells, helper T cells, cytotoxic T cells, CD4^+^/CD8^+^ ratio	None	8
10	Hosseni et al. (2007), Iran	Depression (*n* = 37) Controls (*n* = 15)^c^	Depression: 73% Controls: Not reported	Depression: 39.1 ± 12.3 Controls: 38.2 ± 12	Cases: Hospital referrals Controls: Community	DSM-IV criteria for MDD, with select psychiatric comorbidities and medications excluded.	No immunomodulatory medications or medications that may affect melancholic symptoms for a minimum of 6 months.	Total lymphocyte, CD3, CD19, CD4, CD8, CD16/56	None	7
11	Kanba et al. (1998), Japan	Depression (*n* = 35) Controls (*n* = 36)^b^	Depression: 40% Controls: 56%	Depression: 51 ± 14 Controls: 47 ± 13	Cases: Hospital Outpatient Controls: Not specified	DSM-IV diagnosis of major depressive disorder (MDD) and ≥8 on the Hamilton Depression Rating Scale (HAMD). No medical or neurological disorders. No medication or substance use for 4 weeks.	No medical or neurological disorders. No medication or substance use for 4 weeks.	Lymphocyte, CD2^+^, CD20	None	5
12	Landmann et al. (1997), Switzerland	Depression (*n* = 23) Controls (*n* = 23)^b^	Depression: 61% Controls: 61%	Depression median: 43 Controls median: 44	Cases: Clinic outpatients Controls: Community	DSM-III-R criteria for major depressive episode and a score of ≥20 on the HAMD. No medical disorder or substance abuse. No immunomodulatory medications.	No history of depression. No medication. ZAD score <40.	Leukocytes, granulocytes, lymphocytes, monocytes, HLA-DR, CD14	None	5
13	Lynall et al. (2020), UK	Depression (*n* = 206) Controls (*n* = 77)	Depression: 68% Controls: 69%	Depression mean (IQR): 35.3 (29.7, 42.9) Controls mean (IQR): 32.5 (28.3, 39.1)	Cases: Hospital inpatients Controls: Community	DSM-IV current or past depressive symptoms. No other psychiatric disorder or immunomodulating treatment.	No medical or psychiatric disorder. No history of antidepressant medication. No current immunomodulatory medication. HAMD score <7.	RBC, platelets, basophils, eosinophils, neutrophils, monocytes, CD4^+^, CD8^+^, B cells, NK cells	Age, sex, BMI, recent infection, tobacco use	8
14	Maes et al. (1992a), Belgium	Minor depression (*n* = 28) Simple major depression (*n* = 51) Melancholia (*n* = 27) Controls (*n* = 19)	Minor depression: 79% Simple major depression: 75% Melancholia: 59% Controls: 63%	Minor depression: 42.4 ± 2.7 Simple major depression: 44.9 ± 1.8 Melancholia: 52.9 ± 2.2 Control: 41.3 ± 3.1	Cases: Hospital inpatient Controls: Staff or family members	DSM-II diagnosis of minor depression, major depression with or without melancholia. Select medications excluded. Free from chronic and acute medical illnesses that influence immune function and drugs known to affect immune apparatus.	No medical or psychiatric disorders. No psychotropic medication for 1 year prior. No alcohol abuse. BDI score <9, ZDA score <40, State Trait Anxiety Inventory (STAI) score <40. Free from chronic and acute medical illnesses that influence immune function and drugs known to affect immune apparatus.	CD19^+^, CD20^+^, CD21^+^, HLADR^+^	None	5
15	Maes et al. (1992b), Belgium	Minor depression (*n* = 14) Major depression (*n* = 12) Melancholia (*n* = 12) Controls (*n* = 10)	Sex not reported	Minor depression group mean ± SEM: 36.0 ± 2.7 Simple major depression group mean ± SEM: 41.9 ± 4.0 Melancholia group mean ± SEM: 49.9 ± 4.4 Control group mean ± SEM: 43.7 ± 4.1	Cases: Hospital inpatients Controls: Community	DSM-III criteria for respective disorders. No bipolar depression or other axis I diagnoses. Select medications and treatments excluded. No medical disorders related to alternations in immune function. No history of infectious disease.	No medical or psychiatric disorders. Free of any medication. Not regular drinkers. No history of infectious disease.	WBC, lymphocytes, monocytes, granulocytes, CD3^+^, MHC HLA-DR, CD25^−^, CD19^+^, CD4^+^, CD8^+^, CD4/CD8 ratio, CD4^+^CD45RA, CD4^+^CD45RA^+^	None	5
16	Maes et al. (1992c), Belgium	Minor depression (*n* = 28) Simple major depression (*n* = 48) Melancholia (*n* = 25) Controls (*n* = 21)	Minor depression: 71% Simple major depression: 79% Melancholia: 56% Controls: 57%	Minor depression group mean ± SEM: 42.1 ± 2.5 Simple major depression group mean ± SEM: 44.7 ± 1.8 Melancholia group mean ± SEM: 52.4 ± 2.3 Control group mean ± SEM: 42.7 ± 2.9	Cases: Hospital inpatients Controls: Community	DSM-II criteria for respective disorder. No bipolar depression or other axis I diagnoses. Select medications and treatments excluded. No medical disorders related to alternations in immune function. No history of infectious disease.	No medical disorders related to alternations in immune function. No history of infectious disease. Free of any psychotropic drugs for at least 1 year. No alcohol abuse. No somatic or mental illness. BDI score <9. ZDA score <40. STAI score <40.	CD4^+^, CD4^+^CD45^+^, CD4^+^CD45^−^, CD8^+^, CD8^+^CD57^+^, CD8^+^CD57^−^, CD4/CD8 ratio	Age	5
17	Maes et al. (1993a), Belgium	Minor depression (*n* = 20) Simple major depression (*n* = 29) Melancholic (*n* = 14) Controls (*n* = 37)	Minor depression: 70% Simple major depression: 76% Melancholic: 57% Controls: 54%	Minor depression: 44.3 ± 2.9 Simple major depression: 47.1 ± 2.5 Melancholic: 52.6 ± 3.6 Controls: 44.1 ± 1.8	Cases: Hospital inpatients Controls: Staff and family members	DSM-III criteria for respective disorders. No immunomodulatory drugs in the last 7 days or other mental health comorbidities. Select other medications and treatments excluded. No heavy smokers.	No medical or psychiatric illness or depressive symptoms. No medications for at least 1 month prior. No regular drinkers or history of psychotropic drug use. BDI score <9. ZAD score <40. No immunomodulatory drugs or heavy smokers.	Leukocytes, lymphocytes, monocytes, neutrophils, CD25^+^, HLA-DR^+^T	Age, gender	8
18	Maes et al. (1993b), Belgium	Minor depression (*n* = 26) Simple major depression (*n* = 43) Melancholic (*n* = 23) Controls (*n* = 22)	Not reported	Minor depression: 42.1 ± 2.7 Simple major depression: 44.9 ± 2.0 Melancholic: 52.9 ± 2.2 Control: 42.4 ± 2.8	Cases: Hospital inpatients Controls: Staff and family members	DSM-III criteria for respective disorders. No immunomodulatory drugs in the last 7 days or other mental health comorbidities. Select other medications and treatments excluded. No heavy smokers.	No medical or psychiatric illness. No regular drinkers or history of psychotropic drug use. BDI score <9. ZAD score <40. STAI < 40. No heavy smokers.	WBC, monocytes CD2^+^, CD7^+^, CD2^+^HLADR^+^, CD7^+^CD25^+^, CD7^−^CD25^+^	Age	5
19	Maes et al. (1994a), USA	Minor depression (*n* = 20) Simple major depression (*n* = 29) Melancholia (*n* = 30) Controls (*n* = 17)	Minor depression: 75% Simple major depression: 72% Melancholia: 70% Controls: 53%	Minor depression: 36.6 ± 2.6 Simple major depression: 43.1 ± 2.1 Melancholia: 52.5 ± 2.5 Controls: 45.6 ± 2.6	Cases: Hospital inpatient Controls: community	DSM-III diagnosis of minor depression, major depression with or without melancholia. No other axis I comorbidities. Select medications and therapies excluded. No major medical illnesses, abnormal laboratory findings, ECG, EEG, or chest X-ray. Free from acute and chronic medical disorders and drugs known to affect immune function.	No medical or psychiatric disorders. No medication/substance use for 1 year prior to study. No regular alcohol use or history of major psychotropic drug use. Beck’s Depression Inventory (BDI) score <9. Zung Depression and Anxiety (ZDA) score <40. Free from acute and chronic medical disorders and drugs known to affect immune function.	Leukocytes, lymphocytes, monocytes, neutrophils, natural killer (NK) cells	Age, sex	7
20	Maes et al. (1994b), Belgium	Minor depression (*n* = 7) Simple major depression (*n* = 18) Melancholic (*n* = 11) Controls (*n* = 13)	Minor depression: 57.1% Simple major depression: 66.7% Melancholic: 72.7% Controls: 38.5%	Minor depression group mean ± SEM: 36.3 ± 3.2 Simple major depression group mean ± SEM: 48.2 ± 2.9 Melancholia group mean ± SEM: 51.3 ± 2.9 Control group mean ± SEM: 49.4 ± 3.9	Cases: Hospital inpatients Controls: Staff and family members	DSM-III criteria for respective conditions. Exclusionary criteria included comorbid psychiatric illness, immunomodulatory illness, select medications and treatments. Heavy smokers (> 25 cigarettes/day) excluded.	Free of medical or psychiatric illness. BDI score <9. ZDA score <40. Drug free for at least one year. No regular drinkers. Heavy smokers (> 25 cigarettes/day) excluded.	Leukocytes, lymphocytes, monocytes, neutrophils, HLA-DR^+^ T cells, CD25^+^ T cells, NK cells	Drug state, sex, age	7
21	Nowak et al. (2019), Argentina	Depression: (*n* = 33) Controls (*n* = 20)^b^	Depression: 76% Controls: 75%	Depression: 36.35 ± 12.75 Controls: 34.5 ± 5.3	Hospital inpatients	DSM-IV criteria for MDD. Hospitalised for suicide attempt within 48 hours of study. No substance abuse or major medical illness. No medications.	No medications.	Monocytes	None	5
22	Patas et al. (2018), Germany	Depression (*n* = 20) Controls (*n* = 20)^f^	Depression: 55% Controls: 55%	Depression median (IQR): 26.5 (30.5-44) Controls median (IQR): 37 [31–46]	Cases: Clinic outpatients Controls: Community	DSM-IV criteria for MDD. Free from psychiatric medication for 8 weeks prior. HAMD score ≥18. No major medical illness or immunomodulatory medication. No heavy drinkers, current adverse life events, recent vaccination.	No history of mood disorders. Quick Inventory of Depressive Symptoms-Self Report score ≤5. No major medical illness or immunomodulatory medication. No heavy drinkers, current adverse life events, recent vaccination.	Granulocytes, monocytes, lymphocytes, T cells, CD4^+^, CD8^+^, B cells, NK cells	None	7
23	Pavon et al. (2006), Mexico	Depression (*n* = 33) Controls (*n* = 33)^e^	Depression: 85% Controls: 85%	Depression: 33.6 ± 10.2 Controls: 32.3 ± 10.8	Cases: Clinic outpatients Controls: Community	DSM-IV criteria for current MDD and score ≥22 on the HAMD. No neurological or other psychiatric (except anxiety) comorbidities, or drug abuse. No medications for minimum three weeks. Low caffeine, tobacco, and alcohol consumption.	No psychiatric disorder and no medications in the last three weeks. Low caffeine, tobacco, and alcohol consumption.	CD3^+^CD4^+^, CD3^+^ CD8^+^, CD3^−^CD16^+^/CD56^+^, CD3^+^CD19^+^	None	8
24	Ravindran et al. (1996), Canada	Depression (*n* = 18) Dysthymia (*n* = 31) Controls (*n* = 23)	Depression: 50% Dysthymia: 58% Controls: 61%	Depression: - female 37.22 ± 2.93 - male 37.88 ± 4.53 Dysthymia: - female 39.61 ± 2.00 - male 43.46 ± 1.34 Controls: - female 35.14 ± 2.22 - male 35.11 ± 3.00	Cases: Hospital outpatients Controls: Community	DSM-IIIR criteria for their respective disorders. Free from other axis I or II disorders. No other major medical disorder requiring medication. No alcohol/substance abuse. Low caffeine intake.	No psychiatric illness or medical disorder requiring medication. No alcohol or substance abuse. Low caffeine intake. BDI score <4.	NK, CD3, CD4, CD8, CD19 cells.	None	5
25	Ravindran et al. (1998), Canada	Depression (*n* = 37) Atypical depression (*n* = 32) Dysthymia (*n* = 46) Atypical dysthymia (*n* = 46) Controls (*n* = 44)	Depression: 54% Atypical depression: 66% Dysthymia: 59% Atypical dysthymia: 55% Controls: 31%	Depression means ± SEM: - female 39.00 ± 2.61 - male 31.41 ± 3.58 Control means ± SEM: - female 37.61 ± 1.60 - male 34.37 ± 1.69	Cases: Hospital outpatients Controls: Not specified	DSM-IIIR/DSM-IV criteria for their respective disorders. Free from other axis I or II disorders. No major medical disorder or immunomodulatory medication. No alcohol/substance abuse. Low caffeine intake.	No psychiatric illness or medical disorder that requires immunomodulatory medication. No alcohol or substance abuse. Low caffeine intake. No history of psychotropic drug use. BDI score <4.	Lymphocytes, NK, CD3, CD8, CD4, CD4/CD8, CD19	Age	5
26	Ravindran et al. (1999), Canada	Depression (*n* = 26) Obsessive-compulsive disorder (OCD; *n* = 26) Controls (*n* = 26)^b^	Depression: 54% OCD: 54% Controls: 54%	Depression: Matched to OCD OCD: 31.33 ± 3.33 Controls: Matched to OCD	Cases: Hospital outpatients Controls: Not specified	DSM-IIIR/DSM-IV criteria for MDD or OCD. Free from other axis I or II disorders. No major medical disorder or immunomodulatory medication. No alcohol/substance abuse.	No psychiatric illness, major medical disorder, or immunomodulatory medication. No alcohol or substance abuse. No history of psychotropic drug use. No alcohol/substance abuse. BDI score <4.	Lymphocytes, CD16^+^/CD56^+^, CD3^+^, CD4^+^, CD8^+^, CD4/CD8, CD19^+^	None	6
27	Robertson et al. (2005), USA	Depressed (*n* = 24) Chronic fatigue syndrome (*n* = 23) Multiple sclerosis (*n* = 22) Controls (*n* = 25)^b^	Depressed: 75% Chronic fatigue syndrome: 73.9% Multiple sclerosis: 77.3% Controls: 76%	Depressed: 35.5 Chronic fatigue syndrome: 38.6 Multiple sclerosis: 39.9 Controls: 37.7	Cases: Hospital outpatients Controls: Community	DSM-III-R diagnosis of major depression. CDC definition for chronic fatigue syndrome. Poser et al. criteria for Multiple sclerosis.	No history of depression, chronic fatigue syndrome, multiple sclerosis or any chronic symptoms required for chronic fatigue syndrome diagnosis. All controls reported to be in good health.	Lymphocytes, CD2^+^ PBL, CD3^+^, CD20^+^, CD56^+^, CD3^+^/CD25^−^, CD3^+^/CD16^−^, CD3^+^/CD25^+^, CD8^+^/HLA-DR^+^, CD8^+^/CD38^+^, CD20^+^/CD5^+^, CD20^+^/CD25^+^, CD20^+^/CD23^+^, CD16^+^/CD3^−^, CD56^+^/CD25^+^, CD56^+^/CD16^−^, CD56^+^/CD2^−^	None	7
28	Rothermundt et al. (2001), Germany	Major depression (*n* = 43) Melancholic depression (*n* = 22) Non-melancholic depression (*n* = 21) Controls (*n* = 43)^b^	Depression: 65% Controls: 65%	Depression: 44.45 ± 9.95 Controls: 44.45 ± 9.95	Cases: Hospital inpatients Controls: Community	DSM-IV, ICD-10, and CIDI criteria for major depressive episode. No major medical illness or immunomodulatory medications.	No major medical illness or immunomodulatory medications.	Leukocytes, Lymphocytes, NK cells	None	7
29	Schiweck et al. (2020), Belgium	Depression (*n* = 153) Controls (*n* = 153)^b^	Depression: 63% Controls: 63%	Depression: 37.76 ± 12.97 Controls: 37.84 ± 12.79	Cases: Not specified Controls: Community	Mini-International Neuropsychiatric Interview diagnosed MDD. No immunomodulatory medication or illness. No recent or current pregnancy.	No psychiatric diagnoses. No immunomodulatory medication or illness. No recent or current pregnancy.	Lymphocytes, T cells, NK cells, B cells. T helper cells, T cytotoxic cells, monocytes, T_h_1, T_h_2, T_h_17, T_reg_ cells, CD3^+^CD4^+^CD45RO	Age, sex, BMI, depression severity, medication status	7
30	Schlatter, Ortuño & Cervera-Enguix (2004), Spain	Melancholia (*n* = 14) Non-melancholic depression (*n* = 28) Controls (*n* = 20)	Melancholia: 57.14% Non-melancholic depression: 60.7% Controls: 50%	Melancholia: 48.0 ± 14.5 Non-melancholic depression: 40.0 ± 5.0 Controls: 35.0 ± 9.2	Cases: Hospital inpatients Controls: Not specified	DSM-IV criteria for respective disorder. Free from any comorbid axis I or II disorders. HAMD score > 7. No immunomodulatory drugs. No pregnant or lactating women.	No immunomodulatory illnesses or medications. No pregnant or lactating women.	CD4^+^, CD8^+^, CD4^+^CD8^+^, CD4^+^/CD8^+^, CD4^+^CD45^+^RA^+^, CD4^+^CD45^+^RO^+^, CD8^+^CD45^+^RA^+^, CD8^+^CD45^+^RO^+^, CD16^+^56^+^	Age	4
31	Seidel et al. (1996), Germany	Depression (*n* = 33) Controls (*n* = 44)	Depression: 63.6% Controls: 63.6%	Depression: 38.8 ± 9.0 Controls: 37.5 ± 10.6	Cases: Hospital inpatients Controls: Community	DSM-III-R criteria for MDD. No immunomodulatory treatment or disorders No comorbid psychiatric diagnoses. No ECT within 1 year prior to hospitalisation. Select medications excluded.	No immunomodulatory disorders.	CD4, CD5, CD8, CD19, CD45RO	None	4
32	Suzuki et al. (2017), USA	Depression (*n* = 54) Controls (*n* = 56)^b^	Depression: 76% Controls: 75%	Depression: 34.3 ± 11.20 Controls: 32.1 ± 10.93	Cases: Hospital clinics Controls: Community	DSM-IV-TR MDD. No psychotropic drug for 3 weeks minimum prior. No major medical or neurological illness, psychosis, traumatic brain injury, history of drug/alcohol abuse within one year prior.	No history of any major psychiatric disorder in first degree relatives. No major medical or neurological illness, psychosis, traumatic brain injury, history of drug/alcohol abuse within one year prior.	CD3^+^, CD4^+^, CD8^+^, central memory, naïve, effector memory, T_h_1, T_h_2, T_h_17, T regulatory (T_reg_) cells, CD3−/CD19−, CD14−/CD20−, HLA-DR+, DCs, HLA-DR−, monocytes, NK cells	BMI, age, sex, batch effect	7
33	Syed et al. (2018), USA	Depression (*n* = 171) Controls (*n* = 64)	Depression: 66% Controls: 47%	Depression: 39.4 ± 11.8 Controls: 45 ± 11.8	Cases: Outpatients clinics Controls: Community	DSM-IV criteria for MDD without psychotic features, >14 on the HAMD, and no history of adequate treatment (4 weeks antidepressant or psychological therapy). Select disorders excluded.	Not taking medications at time of blood sampling.	CD4^+^CD25^+^CD69^+^, CD4^+^CD45RO^+^CD69^−^, CD4^+^CD69^+^CD45RO^−^, CD8^+^CD69^+^, CD19^+^CD69^+^, CD11bCD86^+^	Age, gender, race	5

^a^Age matched.

^b^Age and sex matched.

^c^Age, sex, and socioeconomically matched.

^d^Age, sex, and race matched.

^e^Age, sex, socioeconomic status, and ethnicity matched.

^f^Age, sex, BMI, and smoking status matched.

^g^All smokers, free from symptoms of pulmonary disease.

^h^Quality assessed using Newcastle Ottawa Scale for case-control studies with maximum possible score of eight.

### Meta-analysis of total WBC

Meta-analysis of seven studies comprising 163 depression cases and 105 controls showed an increase in mean total WBC count in cases compared to controls (SMD = 1.07; 95% CI, 0.61–1.53; *P* < 0.01; *I*^2^ = 64%; Supplementary Fig. 1).

### Meta-analysis of myeloid cells

Mean absolute counts of granulocytes, neutrophils, and monocytes were found to be higher in depression cases compared to controls (detailed below). Relative percentage of neutrophils was also higher in cases compared to controls.

#### Granulocytes

Meta-analysis of two studies totalling 34 cases and 32 controls showed higher mean total granulocyte count in depression (SMD = 2.07; 95% CI, 1.45–2.68; *P* < 0.01; *I*^2^ = 0%; Supplementary Fig. 2).

#### Neutrophils

**Fig. 2:**
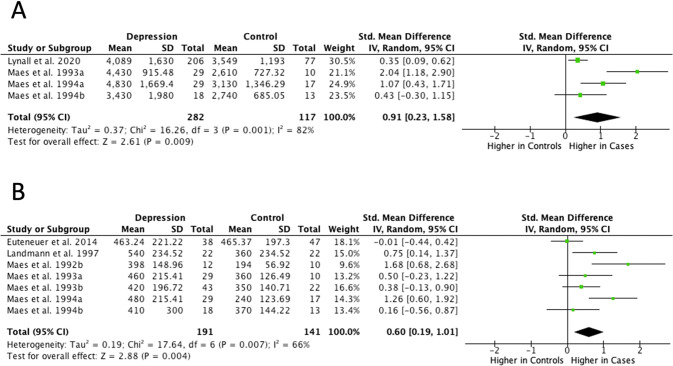
Meta-analysis of mean absolute counts of neutrophils (**A**) and monocytes (**B**) in depression vs controls.

Meta-analysis of four studies with 282 cases and 117 controls showed an increase in mean total neutrophil count in depression (SMD = 0.91; 95% CI, 0.23–1.58; *P* < 0.01; *I*^2^ = 82%; Fig. 2A). Similarly, separate meta-analysis of three studies showed higher relative percentage of neutrophils in depression compared to controls (SMD = 1.90; 95% CI, 0.02–3.78; *P* = 0.05; *I*^2^ = 93%; Supplementary Fig. 3). Results were similar after removing outliers and heterogeneity reduced to 0% (Supplementary Fig. 4).

#### Monocytes (CD14^+^)

Meta-analysis of seven studies with 191 cases and 141 controls showed higher mean absolute monocyte counts in depression (SMD = 0.60; 95% CI, 0.19–1.01; *P* < 0.01; *I*^2^ = 66%; Fig. 2B). However, separate meta-analysis of six studies with 250 cases and 300 controls that reported relative percentage of monocytes showed no difference between groups (SMD = 0.26; 95% CI, −0.08–0.60; *P* = 0.13; *I*^2^ = 65%; Supplementary Fig. 5).

### Meta-analysis of lymphoid cells

Mean absolute CD4^+^ helper T cell, natural killer (NK) cell, B cell, and activated T cell counts were found to be higher in depression cases compared to controls (detailed below). Relative percentage of lymphocytes, Th1, and Th2 were shown to be decreased in cases compared to controls.

#### Total lymphocytes

Meta-analysis of 10 studies comprising 263 cases and 205 controls showed no difference in mean absolute total lymphocyte count between cases and controls (SMD = −0.18; 95% CI, −0.65–0.30; *P* = 0.47; *I*^2^ = 83%; Supplementary Fig. 6). Results were similar after removing outliers and heterogeneity reduced to 58% (Supplementary Fig. 7). However, separate meta-analysis of six studies that reported relative percentage of total lymphocytes showed a decrease in depression cases compared to controls (SMD = −0.41; 95% CI, −0.80–−0.02; *P* = 0.04; *I*^2^ = 67%; Supplementary Fig. 8).

#### T (CD3^+^), helper T (CD4^+^), and cytotoxic T (CD8^+^) cells

**Fig. 3:**
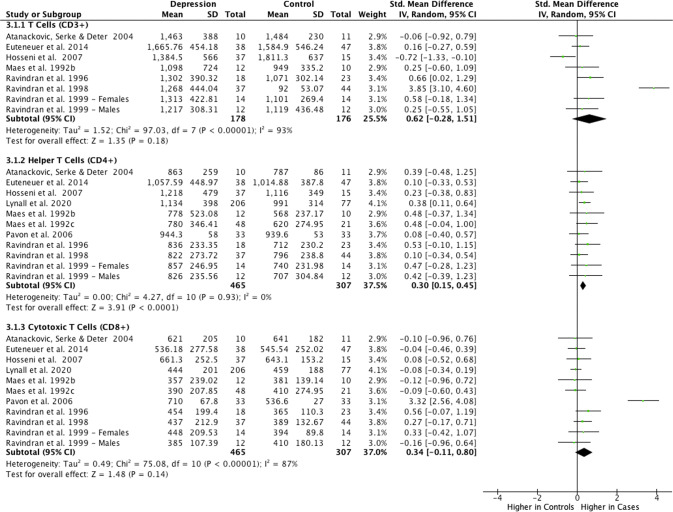
Meta-analysis of mean absolute counts of T cell subsets in depression vs controls.

Meta-analysis of 11 studies totalling 465 cases and 307 controls showed higher CD4^+^ helper T cell count in depression (SMD = 0.30; 95% CI, 0.15–0.45; *P* < 0.01; *I*^2^ = 0%; Fig. 3). However, total CD3^+^ and CD8^+^ T cell count did not differ between groups (Fig. 3). Results were similar when outliers for CD3^+^ and CD8^+^ T cells were excluded from meta-analysis and heterogeneity in meta-analysis decreased for both cell types (Supplementary Fig. 9).

Separate meta-analyses of relative percentages of CD3^+^ T cells, CD4^+^ helper T cells, and CD8^+^ cytotoxic T cells, including outliers, showed no differences between depressed cases and controls (Supplementary Fig. 10). Results were similar when the single extreme CD8^+^ T cell outlier was excluded from meta-analysis and heterogeneity in meta-analysis decreased (Supplementary Fig. 11).

#### Helper T cell subsets: Th1, Th2, and Th17 cells

No studies reported absolute cells counts for Th1, Th2, or Th17 cells, but relative percentage data for Th1 (four studies), Th2 (three studies), and Th17 (four studies) cells was available. Relative percentage of Th1 (SMD = −0.31; 95% CI, −0.49–−0.14; *P* < 0.01; *I*^2^ = 0%) and Th2 (SMD = −0.36; 95% CI, −0.55–−0.17; *P* < 0.01; *I*^2^ = 0%) cells were found to be lower in depression compared to controls (Supplementary Fig. 12). There was no difference in Th17 cells (Supplementary Fig. 12).

#### T Regulatory cells

Meta-analysis of four studies with 233 cases and 316 controls showed no difference in relative percentage of T regulatory cells between groups (SMD = −0.13; 95% CI, −0.30–0.04; *P* = 0.14; *I*^2^ = 3%; Supplementary Fig. 13).

#### NK cells (CD16^+^/CD56^+^)

**Fig. 4:**
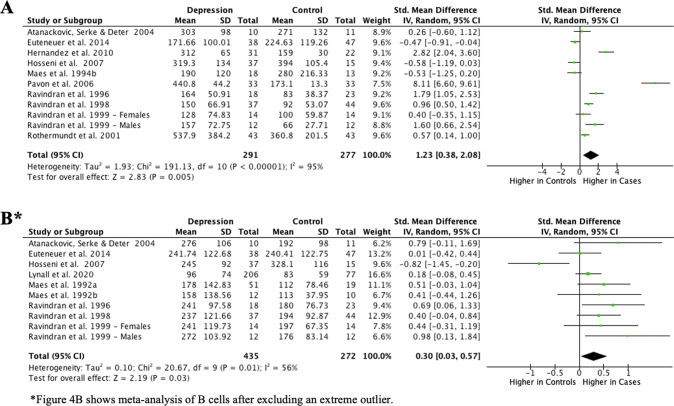
Meta-analysis of mean absolute counts of natural killer cells (**A**) and CD19+ B cells (**B**) in depression vs controls.

Meta-analysis of 11 studies comprising 291 cases and 277 controls showed higher mean absolute NK cell count (SMD = 1.23; 95% CI, 0.38–2.08; *P* < 0.01; *I*^2^ = 95%; Fig. 4A) in cases compared to controls. Results were similar when the single extreme outlier was excluded from meta-analysis and heterogeneity reduced to 95% (Supplementary Fig. 14). However, meta-analysis of eight studies of relative percentage of NK cells showed no difference between groups (SMD = 0.50; 95% CI, −0.23–1.24; *P* = 0.18; *I*^2^ = 95%; Supplementary Fig. 15). Results were similar when the single extreme outlier was excluded from meta-analysis and heterogeneity in meta-analysis decreased (Supplementary Fig. 16).

#### B cells (CD19^+^)

Meta-analysis of 11 studies of mean absolute B cell count with 468 cases and 305 controls showed no difference between groups (SMD = −0.24; 95% CI, −0.88–0.41; *P* = 0.48; *I*^2^ = 93%; Supplementary Fig. 17). However, after excluding the single extreme outlier, meta-analysis of 10 studies with 435 cases and 272 controls showed higher mean absolute B cell count in depression, and heterogeneity in meta-analysis decreased (SMD = 0.30; 95% CI, 0.03–0.57; *P* = 0.03; *I*^2^ = 56%; Fig. 4B).

Meta-analysis of seven studies reporting relative percentage of B cells found no difference between groups (SMD = −0.22; 95% CI, −0.74–0.30; *P* = 0.420 *I*^2^ = 91%; Supplementary Fig. 18). Results were similar when the single extreme outlier was excluded from meta-analysis and heterogeneity dropped to 35% (Supplementary Fig. 19).

#### Activated T cells: CD25^+^ and CD3^+^HLADR^+^

**Fig. 5:**
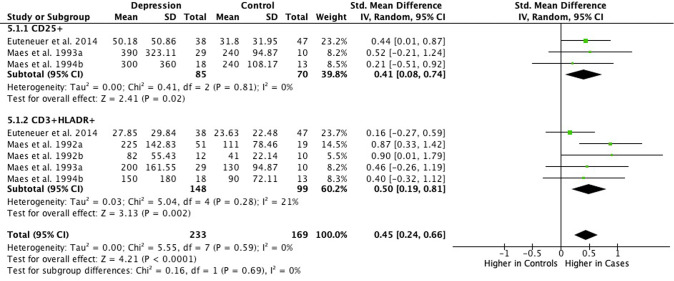
Meta-analysis of mean absolute counts of activated T cells in depression vs controls.

Meta-analysis of eight studies totalling 233 cases and 169 controls showed higher mean absolute cell counts of activated T cells (CD25^+^ and CD3^+^HLADR^+^) in depressed cases compared to controls (SMD = 0.45; 95% CI, 0.24–0.66; *P* < 0.01; *I*^2^ = 0%; Fig. 5). In particular, SMD for mean absolute cell counts of CD25^+^ in depression was 0.41 (95% CI, 0.08–0.74; *P* = 0.02; *I*^2^ = 0%; Fig. 5) and SMD for mean absolute CD3^+^HLADR^+^ cells in depression was 0.50 (95% CI, 0.19–0.81; *P* < 0.01; *I*^2^ = 21%; Fig. 5). However, meta-analysis of seven studies with 371 cases and 160 controls found no difference between groups in relative percentage of activated T cells (SMD = 0.30; 95% CI, −0.26–0.85; *P* = 0.29; I^2^ = 85%; Supplementary Fig. 20).

#### CD4^+^/CD8^+^ ratio

Meta-analysis of seven studies with 130 cases and 133 controls showed no difference in mean absolute CD4^+^/CD8^+^ ratio count between groups (SMD = 0.42; 95% CI, −0.41–1.25; *P* = 0.32; *I*^2^ = 89%; Supplementary Fig. 21). Results were similar when outliers were excluded from meta-analysis and heterogeneity reduced to 21% (Supplementary Fig. 22).

#### Naïve T cells (CD45RA^+^)

Meta-analysis of two studies with 50 cases and 57 controls showed no difference in mean absolute naïve T cell counts between groups (SMD = 0.29; 95% CI, −0.10–0.67; *P* = 0.14; *I*^2^ = 0%; Supplementary Fig. 23). Moreover, meta-analysis of three studies with 36 cases and 41 controls found no difference between groups in relative percentage of naïve T cells (SMD = −0.04; 95% CI, −0.86–0.78; *P* = 0.93; *I*^2^ = 68%; Supplementary Fig. 24).

#### Memory T cells (CD45RO^+^/CD45RA^−^)

Meta-analysis of five studies with 242 cases and 159 controls found no difference between groups in relative percentage of memory T cells (SMD = −0.13; 95% CI, −0.80–0.54; *P* = 0.70; *I*^2^ = 87%; Supplementary Fig. 25).

### Assessment of bias

All 33 studies included in this systematic review had an NOS score of ≥4 out of a maximum of eight, indicating reasonable study quality and relatively low risk of bias (Table 1; Supplementary Table 4). However, two studies scored four stars indicating possible risk of bias and 24 of the 33 studies included were deemed at risk of bias regarding selection of controls, comparability, and/or method of ascertainment for cases and controls.

Publication bias was assessed using Egger’s test (Supplementary Table 5) for all meta-analyses, except for five WBC subsets due to the small number of studies included. There was evidence of publication bias for two subsets, namely studies of mean absolute counts of total lymphocytes (*p* = 0.02) and relative percentage of cytotoxic T cells (*p* = 0.02).

## Discussion

Our systematic review and meta-analysis suggests that depression is associated with alterations in several myeloid and lymphoid cells, including increased mean absolute counts of total WBC, granulocytes, neutrophils, monocytes, CD4^+^ helper T cells, NK cells, CD19^+^ B cells, and CD25^+^ and CD3^+^HLADR^+^ activated T cells. Individuals with depression also displayed increased relative percentage of neutrophils, but decreased relative percentage of lymphocytes, Th1, and Th2 cells, although relatively fewer studies assessed relative percentage of cells. To the best of our knowledge, this is the first systematic review and meta-analysis to consider the peripheral blood cellular immunophenotype of depression, and our findings highlight the potential role of both innate and adaptive immune dysfunction in the aetiology of the illness.

Our results suggest that peripheral blood immune cell counts could be useful biomarkers for the identification of inflammation-related depression subgroups, and for immunological treatment development by informing patient stratification in future clinical trials. We report evidence of leukocytosis, monocytosis, and neutrophilia in depression, which is consistent with previous research implicating innate immune dysfunction in the pathogenesis of this illness [59–61]. In particular, previous studies have reported increased leukocytes and myeloid cells, particularly neutrophils and monocytes, in depression [17, 19, 62]. Furthermore, we provide a novel finding of increased absolute granulocyte counts in depressed cases, further implicating myeloid cells in depression aetiology.

Though myeloid cells are strongly implicated in the data presented, it is clear that lymphoid cells also play an important role. It is known that CD4^+^ helper T cells facilitate cytokine production [63]. We show results for cells that are consistent with cytokine signature and reveal potential cellular sources of cytokine alterations implicated in depression. In addition, we present evidence of elevated NK cell, B cell, and activated T cell absolute counts in depression. Taken together, our results not only provide further evidence of immune dysregulation in depression, but also implicate both the innate and adaptive immune responses in the pathogenesis of the disease.

These findings could be useful for several reasons. First, while the evidence of cytokine abnormalities is well established in depression, immune cell alterations in this illness are relatively less understood. Identifying cellular immunophenotypes in depression may help elucidate a cellular source(s) of these known cytokine alterations. Moreover, cell phenotyping in addition to protein data may prove useful for illness subtyping. This would be particularly beneficial for patient stratification in future immunotherapy RCTs. Our investigation also highlights the need for future investigations to determine the direction of association between immune cell alterations identified and depression. If deemed causal, immune cells may represent suitable treatment targets for major depressive disorder.

We report that depression is associated with a decrease in relative percentage of total lymphocytes, Th1, and Th2 cells and an increase in relative percentage of neutrophils. However, several cell types displaying a large effect in meta-analyses of mean absolute cell counts showed no effect in meta-analyses of changes in relative cell percentage. One possible explanation could be that relative percentages are a holistic representation of the immune landscape at the time of sampling. Unlike total cell count, change in relative percentage of one cell type can be influenced by changes in another cell type(s). For example, our finding of a decrease in the relative percentage of lymphocytes, Th1, and Th2 cells may indicate an absolute decrease in the number of these cells in depressed cases compared to controls, or an absolute increase in the number of a different cell type(s). Therefore, in line with standard practice in immunology we focus on results for mean absolute cell count as primary.

Although our investigation revealed a number of cellular differences between depressed cases and controls, there are also several null findings of interest. Firstly, CD4^+^/CD8^+^ ratio has been reported to be higher in depressed patients [19]. Though our research found a possible trending increase in absolute numbers of this ratio, the confidence intervals overlapped the null. Moreover, no associations were identified for absolute numbers of total lymphocytes, CD3^+^ cells, CD8^+^ cells, T regulatory cells, or naïve T cells. Aside from the possibility of there being no actual differences between groups on these cell types, these lack of associations may also be due to the low number of studies that focused on these cell subsets, the immunophenotyping methods used in these studies, and sample characteristics. Basophils and eosinophils were also of interest to the current investigation, however, only one eligible study reported these cell counts. Therefore, future immunophenotyping research should focus on exploring more detailed levels of the cellular hierarchy in patients with depression.

Strengths of this work include the systematic literature search of two databases which identified 1,286 cases and 991 control participants. All studies were assessed using the validated Newcastle-Ottawa Scale (NOS) for case-control studies and publication bias was evaluated by visual inspection of funnel plots and Egger’s test. While the majority of studies originated from Europe and North America, countries outside these regions were also represented in our final selection. Rigorous inclusion criteria was applied to ensure depressed cases were defined using a validated scale, controls had no lifetime history of psychiatric illness, and immunophenotyping was conducted using an established method.

Limitations of this work include the quality of studies included. Case-control studies are prone to selection bias, as reflected by the NOS scores for many of the included studies. While most of these studies recruited controls from the community, psychiatric assessment was often unblinded and not all studies reported method of control recruitment. Second, meta-analyses of certain cell types, namely granulocytes and naïve T cells, were based on data from only two studies. Although meta-analysis can be carried out using data from a minimum of two studies, standard random-effects meta-analysis methods have been reported to perform poorly when applied to few studies [64–66]. Therefore, while we report a large effect for mean absolute counts of granulocytes and no effect for mean absolute naïve T cell count, these results should be considered in the context of limited data currently available. Overall, compared with studies of circulating immune proteins [1–5], there are fewer studies of immune cell counts in depression at present. Further studies are required, as our results suggest that such studies can provide valuable insights into the nature of immune dysfunction in depression. Moreover, although we conducted a thorough and up-to-date literature search, publication bias, especially from selective non-reporting of null findings, is always a possibility for meta-analysis. We assessed evidence of publication bias using visual inspection of funnel plots and Egger’s test. All but two studies showed no evidence of publication bias. Egger’s test was found to be significant for mean absolute total lymphocyte counts and relative percentages of cytotoxic T cells, indicating possible publication bias in these subsets. Most included studies controlled for confounding (particularly age and sex), but this was not the case for all studies. Therefore, more robust confounding adjustment, for example ethnicity, socio-economic status, medications, physical illness, and/or body mass index, is required in future studies. Considerable heterogeneity between studies can be observed in select analyses, however, after the removal of outliers, *I*^2^ statistic dropped and results remained similar for most analyses. However, it is worth noting that heterogeneity remained high for select cell types (i.e., WBC, neutrophils, monocytes, NK cells). Additionally, this review and respective protocol was not registered prior to analysis. Lastly, by design it is difficult to infer causality from case-control studies, and so future research is needed to examine whether observed associations are likely to be causal. Genetic approaches, such as MR analysis, could be particularly useful for this purpose. Results for many cell types were mixed, possibly due to phenotyping method used and sample characteristics, and so there is a need for the standardisation of immunophenotyping methods in studies of depression.

In summary, depression is associated with altered peripheral blood myeloid and lymphoid cell counts, particularly WBC, granulocytes, neutrophils, monocytes, CD4^+^ helper T cells, NK cells, and activated T cells, and with altered cell relative percentages of neutrophils, lymphocytes, Th1, and Th2 cells. These findings are consistent with a potential role of both innate and adaptive immune dysfunction in the aetiology of depression. Immune cells could be useful biomarkers for illness sub-typing and the identification of inflammation-related depression subgroups for patient stratification in future immunotherapy trials. Further research is required to establish whether and how altered immune cells may causally influence the risk of depression, in consort with changes in cytokines and other immune protein levels/activity.

## Supplementary information

Supplemental Material
