# Supplementary material for: Peripheral blood cellular immunophenotype in depression: a systematic review and meta-analysis
Source: Mol Psychiatry. 2022 Dec 28;28(3):1004–19. doi: 10.1038/s41380-022-01919-7 (PMC10005954; doi:10.1038/s41380-022-01919-7)
Supplement: Supplementary file 1 — Supplemental Material [file 41380_2022_1919_MOESM1_ESM.docx]

**Supplementary Material**

**Foley *et al*. Peripheral blood cellular immunophenotype in depression: A systematic review and meta-analysis**

**Contents**

[1. Search Strategy 3](#_Toc119662807)

[2. Supplementary Tables 4](#_Toc119662808)

[Supplementary Table 1. PRISMA 2020 Checklist 4](#_Toc119662809)

[Supplementary Table 2. Individual immune cell types and cell classifications used in the current investigation 8](#_Toc119662810)

[Supplementary Table 3. Unit harmonisation for studies included in the meta-analysis 12](#_Toc119662811)

[Supplementary Table 4. Bias assessment according to the Newcastle-Ottawa scale for case-control studies 13](#_Toc119662812)

[Supplementary Table 5. Egger’s test results for each cell type, excluding outliers 15](#_Toc119662813)

[3. Supplementary Figures 16](#_Toc119662814)

[Supplementary Figure 1. Meta-analysis of total white blood cell count in cases vs controls 16](#_Toc119662815)

[Supplementary Figure 2. Meta-analysis of mean absolute granulocyte count in cases vs controls 16](#_Toc119662816)

[Supplementary Figure 3. Meta-analysis of neutrophil relative percentage in cases vs controls, including outliers 16](#_Toc119662817)

[Supplementary Figure 4. Meta-analysis of neutrophil relative percentage in cases vs controls, excluding outliers 16](#_Toc119662818)

[Supplementary Figure 5. Meta-analysis of monocyte relative percentage in cases vs controls 17](#_Toc119662819)

[Supplementary Figure 6. Meta-analysis of mean absolute total lymphocyte count in cases vs controls, including outliers 17](#_Toc119662820)

[Supplementary Figure 7. Meta-analysis of mean absolute total lymphocyte count in cases vs controls, excluding outliers 17](#_Toc119662821)

[Supplementary Figure 8. Meta-analysis of total lymphocyte relative percentage in cases vs controls 17](#_Toc119662822)

[Supplementary Figure 9. Meta-analysis of mean absolute counts of T cell subsets in depression vs controls, excluding outliers for CD3^+^ and CD8^+^ T cells 18](#_Toc119662823)

[Supplementary Figure 10. Meta-analysis of relative percentage of T cell subsets in cases vs controls, including outliers for CD8^+^ T cells 19](#_Toc119662824)

[Supplementary Figure 11. Meta-analysis of relative percentage of T cell subsets in cases vs controls, excluding outliers for CD8^+^ T cells 20](#_Toc119662825)

[Supplementary Figure 12. Meta-analysis of relative percentage of helper T cell subsets in cases vs controls 21](#_Toc119662826)

[Supplementary Figure 13. Meta-analysis of relative percentage of T regulatory cells in cases vs controls 21](#_Toc119662827)

[Supplementary Figure 14. Meta-analysis of mean absolute natural killer cell count in cases vs controls, excluding outliers 21](#_Toc119662828)

[Supplementary Figure 15. Meta-analysis of relative percentage of natural killer cells in cases vs controls, including outliers 22](#_Toc119662829)

[Supplementary Figure 16. Meta-analysis of relative percentage of natural killer cells in cases vs controls, excluding outliers 22](#_Toc119662830)

[Supplementary Figure 17. Meta-analysis of mean absolute B cell count in cases vs controls, including outliers 22](#_Toc119662831)

[Supplementary Figure 18. Meta-analysis of B cell relative percentage in cases vs controls, including outliers 23](#_Toc119662832)

[Supplementary Figure 19. Meta-analysis of B cell relative percentage in cases vs controls, excluding outliers 23](#_Toc119662833)

[Supplementary Figure 20. Meta-analysis of relative percentage of activated T cell subsets in cases vs controls 23](#_Toc119662834)

[Supplementary Figure 21. Meta-analysis of mean absolute CD4^+^/CD8^+^ ratio counts in cases vs controls, including outliers 24](#_Toc119662835)

[Supplementary Figure 22. Meta-analysis of mean absolute CD4^+^/CD8^+^ ratio counts in cases vs controls, excluding outliers 24](#_Toc119662836)

[Supplementary Figure 23. Meta-analysis of mean absolute naïve T cell counts in cases vs controls 24](#_Toc119662837)

[Supplementary Figure 24. Meta-analysis of relative percentage of naïve T cells in cases vs controls 24](#_Toc119662838)

[Supplementary Figure 25. Meta-analysis of relative percentage of memory T cells in cases vs controls 25](#_Toc119662839)

# **Search Strategy**

**PubMed Search**

Limits: Humans, English

(("Depression"[Mesh] OR "Depressive Disorder"[Mesh] OR "Depressive Disorder, Major"[Mesh]) AND ("Leukocyte Count"[Mesh] OR "Flow Cytometry"[Mesh] OR "Immunophenotyping"[Mesh] OR peripheral blood mononuclear cell OR mass cytometry OR immunophenotype))

**PsycINFO Search**

Limits: Humans, English

(depression OR depressive disorder OR major depressive disorder) AND (leukocyte count OR flow cytometry OR mass cytometry OR immunophenotype OR immunophenotyping OR peripheral blood mononuclear cell)

# **Supplementary Tables**

## *Supplementary Table 1.* PRISMA 2020 Checklist

| **Section and Topic** | **Item #** | **Checklist item** | **Location where item is reported** |
| --- | --- | --- | --- |
| **TITLE** | | | |
| Title | 1 | Identify the report as a systematic review. | p. 1 |
| **ABSTRACT** | | | |
| Abstract | 2 | See the PRISMA 2020 for Abstracts checklist. | p. 2 |
| **INTRODUCTION** | | | |
| Rationale | 3 | Describe the rationale for the review in the context of existing knowledge. | p. 4 - 5 |
| Objectives | 4 | Provide an explicit statement of the objective(s) or question(s) the review addresses. | p. 5 |
| **METHODS** | | | |
| Eligibility criteria | 5 | Specify the inclusion and exclusion criteria for the review and how studies were grouped for the syntheses. | p. 5 - 6 |
| Information sources | 6 | Specify all databases, registers, websites, organisations, reference lists and other sources searched or consulted to identify studies. Specify the date when each source was last searched or consulted. | p. 5 |
| Search strategy | 7 | Present the full search strategies for all databases, registers and websites, including any filters and limits used. | Supplementary Material p. 3 |
| Selection process | 8 | Specify the methods used to decide whether a study met the inclusion criteria of the review, including how many reviewers screened each record and each report retrieved, whether they worked independently, and if applicable, details of automation tools used in the process. | p. 5 - 6 |
| Data collection process | 9 | Specify the methods used to collect data from reports, including how many reviewers collected data from each report, whether they worked independently, any processes for obtaining or confirming data from study investigators, and if applicable, details of automation tools used in the process. | p. 6 |
| Data items | 10a | List and define all outcomes for which data were sought. Specify whether all results that were compatible with each outcome domain in each study were sought (e.g. for all measures, time points, analyses), and if not, the methods used to decide which results to collect. | p. 6 |
|  | 10b | List and define all other variables for which data were sought (e.g. participant and intervention characteristics, funding sources). Describe any assumptions made about any missing or unclear information. | p. 6 |
| Study risk of bias assessment | 11 | Specify the methods used to assess risk of bias in the included studies, including details of the tool(s) used, how many reviewers assessed each study and whether they worked independently, and if applicable, details of automation tools used in the process. | p. 7 |
| Effect measures | 12 | Specify for each outcome the effect measure(s) (e.g. risk ratio, mean difference) used in the synthesis or presentation of results. | p. 7 |
| Synthesis methods | 13a | Describe the processes used to decide which studies were eligible for each synthesis (e.g. tabulating the study intervention characteristics and comparing against the planned groups for each synthesis (item #5)). | p. 7 |
|  | 13b | Describe any methods required to prepare the data for presentation or synthesis, such as handling of missing summary statistics, or data conversions. | p. 7 |
|  | 13c | Describe any methods used to tabulate or visually display results of individual studies and syntheses. | p. 7 |
|  | 13d | Describe any methods used to synthesize results and provide a rationale for the choice(s). If meta-analysis was performed, describe the model(s), method(s) to identify the presence and extent of statistical heterogeneity, and software package(s) used. | p. 7 |
|  | 13e | Describe any methods used to explore possible causes of heterogeneity among study results (e.g. subgroup analysis, meta-regression). | p. 7 |
|  | 13f | Describe any sensitivity analyses conducted to assess robustness of the synthesized results. | p. 7 |
| Reporting bias assessment | 14 | Describe any methods used to assess risk of bias due to missing results in a synthesis (arising from reporting biases). | p. 7 |
| Certainty assessment | 15 | Describe any methods used to assess certainty (or confidence) in the body of evidence for an outcome. | p. 7 |
| **RESULTS** | | |  |
| Study selection | 16a | Describe the results of the search and selection process, from the number of records identified in the search to the number of studies included in the review, ideally using a flow diagram. | p. 8 & Figure 1 |
|  | 16b | Cite studies that might appear to meet the inclusion criteria, but which were excluded, and explain why they were excluded. | p. 8 |
| Study characteristics | 17 | Cite each included study and present its characteristics. | p. 8 & Table 1 |
| Risk of bias in studies | 18 | Present assessments of risk of bias for each included study. | Table 1 & Supplementary Table 4 |
| Results of individual studies | 19 | For all outcomes, present, for each study: (a) summary statistics for each group (where appropriate) and (b) an effect estimate and its precision (e.g. confidence/credible interval), ideally using structured tables or plots. | p. 8 - 21 & Table 1 |
| Results of syntheses | 20a | For each synthesis, briefly summarise the characteristics and risk of bias among contributing studies. | p. 8 - 21 & Table 1 & Supplementary Tables 4 & 5 |
|  | 20b | Present results of all statistical syntheses conducted. If meta-analysis was done, present for each the summary estimate and its precision (e.g. confidence/credible interval) and measures of statistical heterogeneity. If comparing groups, describe the direction of the effect. | p. 8 & 18 - 21 |
|  | 20c | Present results of all investigations of possible causes of heterogeneity among study results. | p. 8 & 18 - 21 |
|  | 20d | Present results of all sensitivity analyses conducted to assess the robustness of the synthesized results. | p. 8 & 18 - 21 |
| Reporting biases | 21 | Present assessments of risk of bias due to missing results (arising from reporting biases) for each synthesis assessed. | p. 21 & Table 1 & Supplementary Tables 4 & 5 |
| Certainty of evidence | 22 | Present assessments of certainty (or confidence) in the body of evidence for each outcome assessed. | p. 8 & 18 - 21 |
| **DISCUSSION** | | |  |
| Discussion | 23a | Provide a general interpretation of the results in the context of other evidence. | p. 21 - 25 |
|  | 23b | Discuss any limitations of the evidence included in the review. | p. 24 - 25 |
|  | 23c | Discuss any limitations of the review processes used. | p. 24 - 25 |
|  | 23d | Discuss implications of the results for practice, policy, and future research. | p. 23 - 25 |
| **OTHER INFORMATION** | | |  |
| Registration and protocol | 24a | Provide registration information for the review, including register name and registration number, or state that the review was not registered. | p. 25 |
|  | 24b | Indicate where the review protocol can be accessed, or state that a protocol was not prepared. | p. 25 |
|  | 24c | Describe and explain any amendments to information provided at registration or in the protocol. | N/a |
| Support | 25 | Describe sources of financial or non-financial support for the review, and the role of the funders or sponsors in the review. | p. 25 - 26 |
| Competing interests | 26 | Declare any competing interests of review authors. | p. 26 |
| Availability of data, code and other materials | 27 | Report which of the following are publicly available and where they can be found: template data collection forms; data extracted from included studies; data used for all analyses; analytic code; any other materials used in the review. | Reported throughout manuscript. |

## *Supplementary Table 2.* Individual immune cell types and cell classifications used in the current investigation

| **Cell Class** | | **Individual Cell Types** |
| --- | --- | --- |
| White Blood Cells (WBC) | | Leukocytes  Leucocytes  White blood cells (WBC) |
| Myeloid Cells | Granulocytes | Granulocytes |
|  | Neutrophils | Neutrophils |
|  | Eosinophils | Eosinophils |
|  | Basophils | Basophils |
|  | Monocytes | Monocytes  CD14^+^ |
| Lymphoid Cells | Total Lymphocytes | Total lymphocytes  Lymphocytes |
|  | T Cell Subsets | T Cells  T Lymphocytes  CD3^+^  CD4^+^CD8^+^  CD4/CD8 |
|  |  | T Helper cell  CD4^+^ Lymphocytes  CD4^+^  CD3^+^CD4^+^  CD3^+^CD4^+^CD8^-^ (Th)  CD3^+^/CD4^+^ |
|  |  | T cytotoxic cells  T suppressor  Suppressor T cells  CD8^+^ Lymphocytes  CD8^+^ T cells  CD8^+^  CD3^+^CD4^-^CD8^+^ (Tc)  CD3^+^CD8^+^  CD3^+^/CD8^+^ |
|  | Helper T Subsets | T_h_1 cells  T helper 1 cells  CD3^+^CD4^+^IFN-y^+^  CD4^+^IFN-y^+^ |
|  |  | T_h_2 cells  T helper 2 cells  CD3^+^CD4^+^IL-4^+^  CD4^+^IL-4^+^ |
|  |  | T_h_17 cells  T helper 17 cells  CD3^+^CD4^+^IL-17A^+^  CD4^+^IL-17A^+^ |
|  | T Regulatory cells | T_reg_ Cells  Natural T regulatory cells  CD3^+^CD4^+^CD25^high^FoxP3^+^  CD4^+^CD25^high^Foxp3^+^ |
|  | Natural Killer (NK) Cells | NK Cells  CD56^+^ Lymphocytes  CD16^+^/56^+^  CD16^+^CD56^+^  CD3^-^CD16/CD56^+^  CD3^-^CD56^+^  CD56^hi^NK cells  CD16^hi^NK cells  NKC  NKH Cells |
|  | B cells | B cells  B Lymphocytes  CD19^+^  CD3^-^CD19^+^ |
|  | Activated T Cells | Activated T cells  CD25^+^  CD3^+^CD25^+^  CD4^+^CD25^+^CD69^+^ |
|  |  | HLA-DR^+^ Lymphocytes  HLA-DR^+^T  CD2^+^HLADR^+^  CD3^+^HLA-DR^+^  CD3^+^/HLA-DR^+^  CD8^+^/CD38^+^  MHC HLA-DR  Anti-HLA-DR |
|  | CD4^+^/CD8^+^ Ratio | CD4^+^/CD8^+^ Ratio  Helper/Suppressor Ratio |
|  | Naïve T Cells | Naïve T cells  Naïve T helper  Naïve Cytotoxic T cells  CD45RA^+^ Lymphocytes  CD45RA^+^  CD3^+^CD45RA^+^  CD4^+^CD45^+^RA^+^  CD4^+^/CD45RA^+^  CD8^+^CD45^+^RA^+^  CD3^+^CD4^+^CD45RA^+^  CD3^+^CD8^+^CD45RA^+^ |
|  | Memory T Cells | Memory T cells  Memory Helper T  Memory Cytotoxic T cells CD45RO^+^/CD45RA^-^  CD3^+^CD45RO^+^  CD4^+^CD45^+^RO^+^  CD4^+^/CD45^+^RO^+^  CD4^+^CD45RO^+^CD69^-^  CD8^+^CD45^+^RO^+^  CD8^+^/CD45RO^+^  CD3^+^CD4^+^CD45RA^-^CD3^+^CD8^+^CD45RA^-^ |

## *Supplementary Table 3.* Unit harmonisation for studies included in the meta-analysis

| **No.** | **Study ID, country** | **Units Reported** | **Conversion Details** |
| --- | --- | --- | --- |
|  |  |  |  |
| 1 | Atanackovic, Serke & Deter (2004), Germany | /μl | No conversion necessary |
| 2 | Başterzi et al. (2010), Turkey | % only | No conversion necessary |
| 3 | Becking et al. (2018), The Netherlands, Germany, Belgium | % only | No conversion necessary |
| 4 | Eutaneuer et al. (2014), Germany | /μl | No conversion necessary |
| 5 | Ghosh et al. (2020), India | % only | No conversion necessary |
| 6 | Grosse et al. (2016a), the Netherlands | % only | No conversion necessary |
| 7 | Grosse et al. (2016b), Germany | % only | No conversion necessary |
| 8 | Hasselmann et al. (2018), Germany | Not included in meta-analysis | |
| 9 | Hernandez et al. (2010), Mexico | cells/ml | No conversion necessary |
| 10 | Hosseni et al. (2007), Iran | cell/ml | No conversion necessary |
| 11 | Kanba et al. (1998), Japan | mm^3^ | No conversion necessary |
| 12 | Landmann et al. (1997), Switzerland | 10/l | Values multiplied by 1,000 |
| 13 | Lynall et al. (2020), UK | 10^3^/µL | Values multiplied by 1,000 |
| 14 | Maes et al. (1992a), Belgium | x10^9/l | Values multiplied by 1,000 |
| 15 | Maes et al. (1992b), Belgium | cells/mm^3^ | No conversion necessary |
| 16 | Maes et al. (1992c), Belgium | x10^9/l | Values multiplied by 1,000 |
| 17 | Maes et al. (1993a), Belgium | x10^9/l | Values multiplied by 1,000 |
| 18 | Maes et al. (1993b), Belgium | x10^9/l | Values multiplied by 1,000 |
| 19 | Maes et al. (1994a), USA | x10^9/l | Values multiplied by 1,000 |
| 20 | Maes et al. (1994b), Belgium | x10^9/l | No conversion necessary |
| 21 | Nowak et al. (2019), Argentina | Not included in meta-analysis | |
| 22 | Patas et al. (2018), Germany | Not included in meta-analysis | |
| 23 | Pavon et al. (2006), Mexico | pg/ml | No conversion necessary |
| 24 | Ravindran et al. (1996), Canada | giga/l | Values multiplied by 1,000 |
| 25 | Ravindran et al. (1998), Canada | giga/l | Values multiplied by 1,000 |
| 26 | Ravindran et al. (1999), Canada | giga/l | Values multiplied by 1,000 |
| 27 | Robertson et al. (2005), USA | Not included in meta-analysis | |
| 28 | Rothermundt et al. (2001), Germany | /μl | No conversion necessary |
| 29 | Schiweck et al. (2020), Belgium | Not included in meta-analysis | |
| 30 | Schlatter, Ortuño & Cervera-Enguix (2004), Spain | Not reported | No conversion deemed necessary |
| 31 | Seidel et al. (1996), Germany | cells/nl^-1^ | No conversion necessary |
| 32 | Suzuki et al. (2017), USA | Not included in meta-analysis | |
| 33 | Syed et al. (2018), USA | % only | No conversion necessary |

## *Supplementary Table 4.* Bias assessment according to the Newcastle-Ottawa scale for case-control studies

| **No.** | **Authors (year), country** | **Selection** | | | | **Comparability** | **Outcome** | | **NOS quality score**  **(max 8)** |
| --- | --- | --- | --- | --- | --- | --- | --- | --- | --- |
|  |  | **Is case definition adequate?** | **Representativeness of cases** | **Selection of controls** | **Definition of controls** | **Comparability of cohorts on basis of design or analysis controlled for confounders** | **Ascertainment of exposure** | **Same method of ascertainment for cases and controls** |  |
| 1 | Atanackovic, Serke & Deter (2004), Germany | 1 | 1 | 1 | 1 | 0 | 1 | 0 | 5 |
| 2 | Başterzi et al. (2010), Turkey | 1 | 0 | 0 | 1 | 1 | 1 | 1 | 5 |
| 3 | Becking et al. (2018), The Netherlands, Germany, Belgium | 1 | 1 | 1 | 1 | 2 | 1 | 0 | 7 |
| 4 | Eutaneuer et al. (2014), Germany | 1 | 1 | 1 | 1 | 2 | 0 | 1 | 7 |
| 5 | Ghosh et al. (2020), India | 1 | 1 | 1 | 1 | 1 | 1 | 0 | 6 |
| 6 | Grosse et al. (2016a), the Netherlands | 1 | 1 | 1 | 1 | 2 | 1 | 0 | 7 |
| 7 | Grosse et al. (2016b), Germany | 1 | 1 | 1 | 1 | 2 | 1 | 0 | 7 |
| 8 | Hasselmann et al. (2018), Germany | 1 | 1 | 0 | 1 | 2 | 1 | 1 | 7 |
| 9 | Hernandez et al. (2010), Mexico | 1 | 1 | 1 | 1 | 2 | 1 | 1 | 8 |
| 10 | Hosseni et al. (2007), Iran | 1 | 1 | 1 | 1 | 2 | 1 | 0 | 7 |
| 11 | Kanba et al. (1998), Japan | 1 | 0 | 0 | 1 | 2 | 1 | 0 | 5 |
| 12 | Landmann et al. (1997), Switzerland | 1 | 1 | 0 | 1 | 2 | 0 | 0 | 5 |
| 13 | Lynall et al. (2020), UK | 1 | 1 | 1 | 1 | 2 | 1 | 1 | 8 |
| 14 | Maes et al. (1992a), Belgium | 1 | 1 | 1 | 1 | 0 | 1 | 0 | 5 |
| 15 | Maes et al. (1992b), Belgium | 1 | 1 | 1 | 1 | 0 | 1 | 0 | 5 |
| 16 | Maes et al. (1992c), Belgium | 1 | 1 | 1 | 1 | 0 | 1 | 0 | 5 |
| 17 | Maes et al. (1993a), Belgium | 1 | 1 | 1 | 1 | 2 | 1 | 1 | 8 |
| 18 | Maes et al. (1993b), Belgium | 1 | 1 | 1 | 1 | 0 | 1 | 0 | 5 |
| 19 | Maes et al. (1994a), USA | 1 | 1 | 1 | 1 | 2 | 1 | 0 | 7 |
| 20 | Maes et al. (1994b), Belgium | 1 | 1 | 1 | 1 | 2 | 1 | 0 | 7 |
| 21 | Nowak et al. (2019), Argentina | 1 | 0 | 0 | 1 | 2 | 1 | 0 | 5 |
| 22 | Patas et al. (2018), Germany | 1 | 1 | 1 | 1 | 2 | 1 | 0 | 7 |
| 23 | Pavon et al. (2006), Mexico | 1 | 1 | 1 | 1 | 2 | 1 | 1 | 8 |
| 24 | Ravindran et al. (1996), Canada | 1 | 1 | 1 | 1 | 0 | 1 | 0 | 5 |
| 25 | Ravindran et al. (1998), Canada | 1 | 1 | 0 | 1 | 1 | 1 | 0 | 5 |
| 26 | Ravindran et al. (1999), Canada | 1 | 1 | 0 | 1 | 2 | 1 | 0 | 6 |
| 27 | Robertson et al. (2005), USA | 1 | 1 | 1 | 1 | 2 | 1 | 0 | 7 |
| 28 | Rothermundt et al. (2001), Germany | 1 | 1 | 1 | 1 | 2 | 1 | 0 | 7 |
| 29 | Schiweck et al. (2020), Belgium | 1 | 1 | 1 | 1 | 2 | 0 | 1 | 7 |
| 30 | Schlatter, Ortuño & Cervera-Enguix (2004), Spain | 1 | 1 | 0 | 1 | 0 | 1 | 0 | 4 |
| 31 | Seidel et al. (1996), Germany | 1 | 1 | 0 | 1 | 0 | 1 | 0 | 4 |
| 32 | Suzuki et al. (2017), USA | 1 | 1 | 1 | 1 | 2 | 0 | 1 | 7 |
| 33 | Syed et al. (2018), USA | 1 | 1 | 0 | 1 | 2 | 0 | 0 | 5 |

## *Supplementary Table 5.* Egger’s test results for each cell type, excluding outliers

| **Cell type** | **Egger’s test result**  **t (p-value)** | |
| --- | --- | --- |
|  | **Mean absolute** | **Relative percentage** |
| White blood cell count | 0.49 (0.64) | - |
| Granulocytes | No. of studies too  small to test | No. of studies too  small to test |
| Neutrophils | 1.84 (0.21) | No. of studies too  small to test |
| Monocytes | 2.46 (0.06) | 1.26 (0.28) |
| Total lymphocytes | **2.87 (0.02)** | -2.70 (0.05) |
| T cells (CD3^+^) | 0.22 (0.83) | 0.80 (0.47) |
| Helper T cells (CD4^+^) | 0.53 (0.61) | 1.25 (0.25) |
| Cytotoxic T cells (CD8^+^) | 0.53 (0.61) | **-2.98 (0.02)** |
| T_h_1 cells | - | -1.84 (0.21) |
| T_h_2 cells | - | -0.38 (0.77) |
| T_h_17 cells | - | 0.72 (0.54) |
| T regulatory cells | - | -1.38 (0.30) |
| NK cells (CD16^+^/CD56^+^) | 1.08 (0.31) | 0.44 (0.68) |
| B cells (CD19^+^) | 0.93 (0.38) | 0.91 (0.40) |
| CD25^+^ | -0.37 (0.78) | No. of studies too small  to test |
| CD3^+^HLADR^+^ | 1.14 (0.34) | 2.79 (0.07) |
| CD4^+^/CD8^+^ ratio | 1.37 (0.26) | - |
| Naïve T cells (CD45RA^+^) | No. of studies too  small to test | 6.22 (0.10) |
| Memory T cells (CD45RO^+^/CD45RA^-^) | - | - 1. (0.22) |

# **Supplementary Figures**

## *Supplementary Figure 1.* Meta-analysis of total white blood cell count in cases vs controls


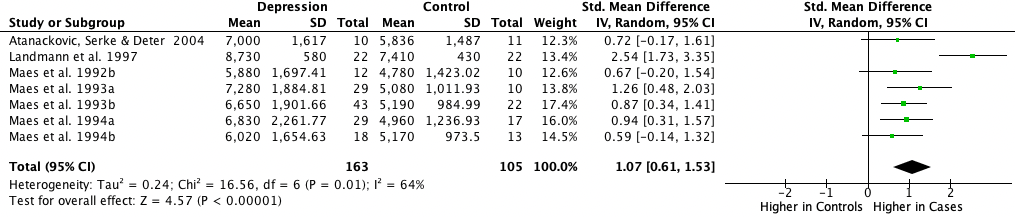


## *Supplementary Figure 2.* Meta-analysis of mean absolute granulocyte count in cases vs controls


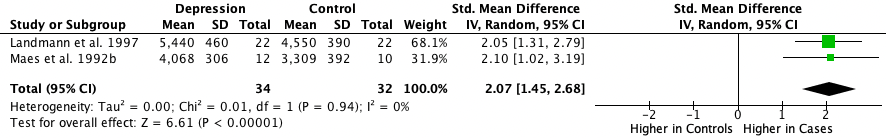


## *Supplementary Figure 3.* Meta-analysis of neutrophil relative percentage in cases vs controls, including outliers


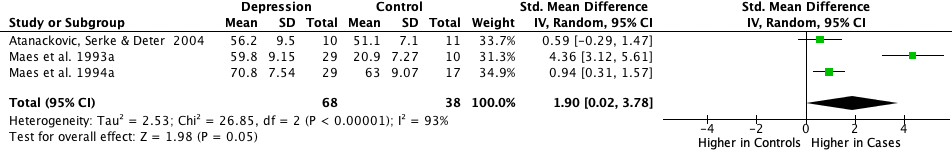


## *Supplementary Figure 4.* Meta-analysis of neutrophil relative percentage in cases vs controls, excluding outliers


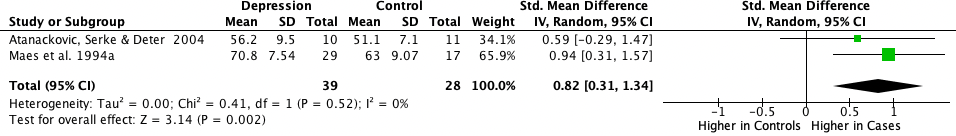


## *Supplementary Figure 5.* Meta-analysis of monocyte relative percentage in cases vs controls


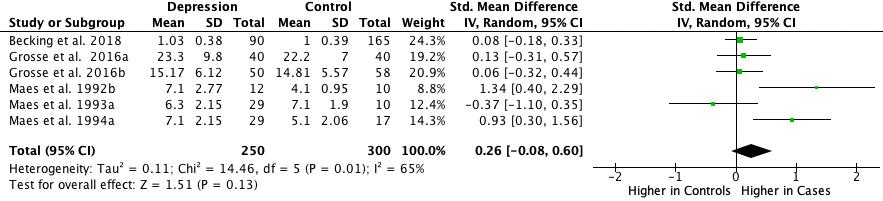


## *Supplementary Figure 6.* Meta-analysis of mean absolute total lymphocyte count in cases vs controls, including outliers


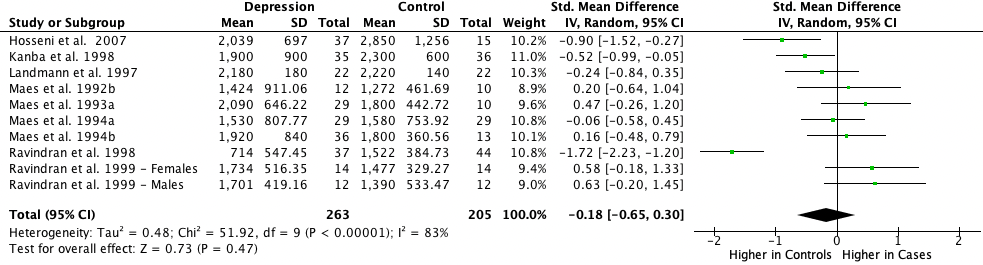


## *Supplementary Figure 7.* Meta-analysis of mean absolute total lymphocyte count in cases vs controls, excluding outliers


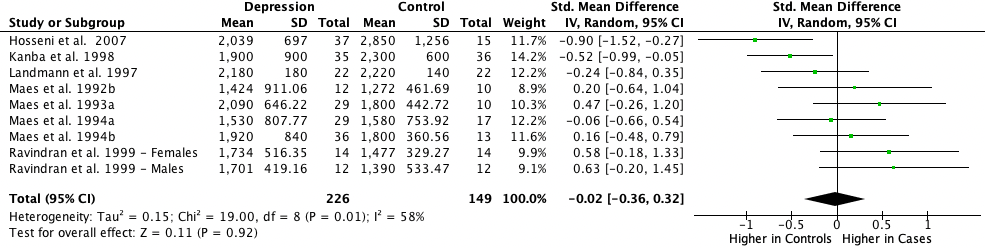


*Supplementary Figure 8*. Meta-analysis of total lymphocyte relative percentage in cases vs controls


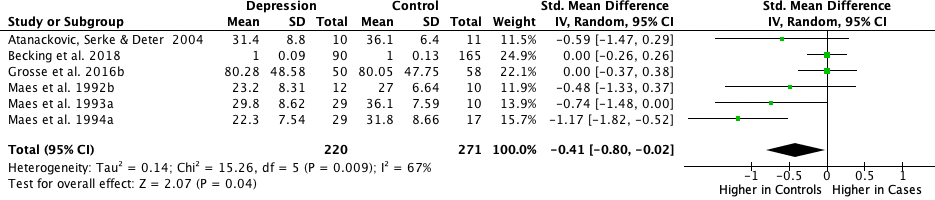


## *Supplementary Figure 9*. Meta-analysis of mean absolute counts of T cell subsets in depression vs controls, excluding outliers for CD3^+^ and CD8^+^ T cells

*
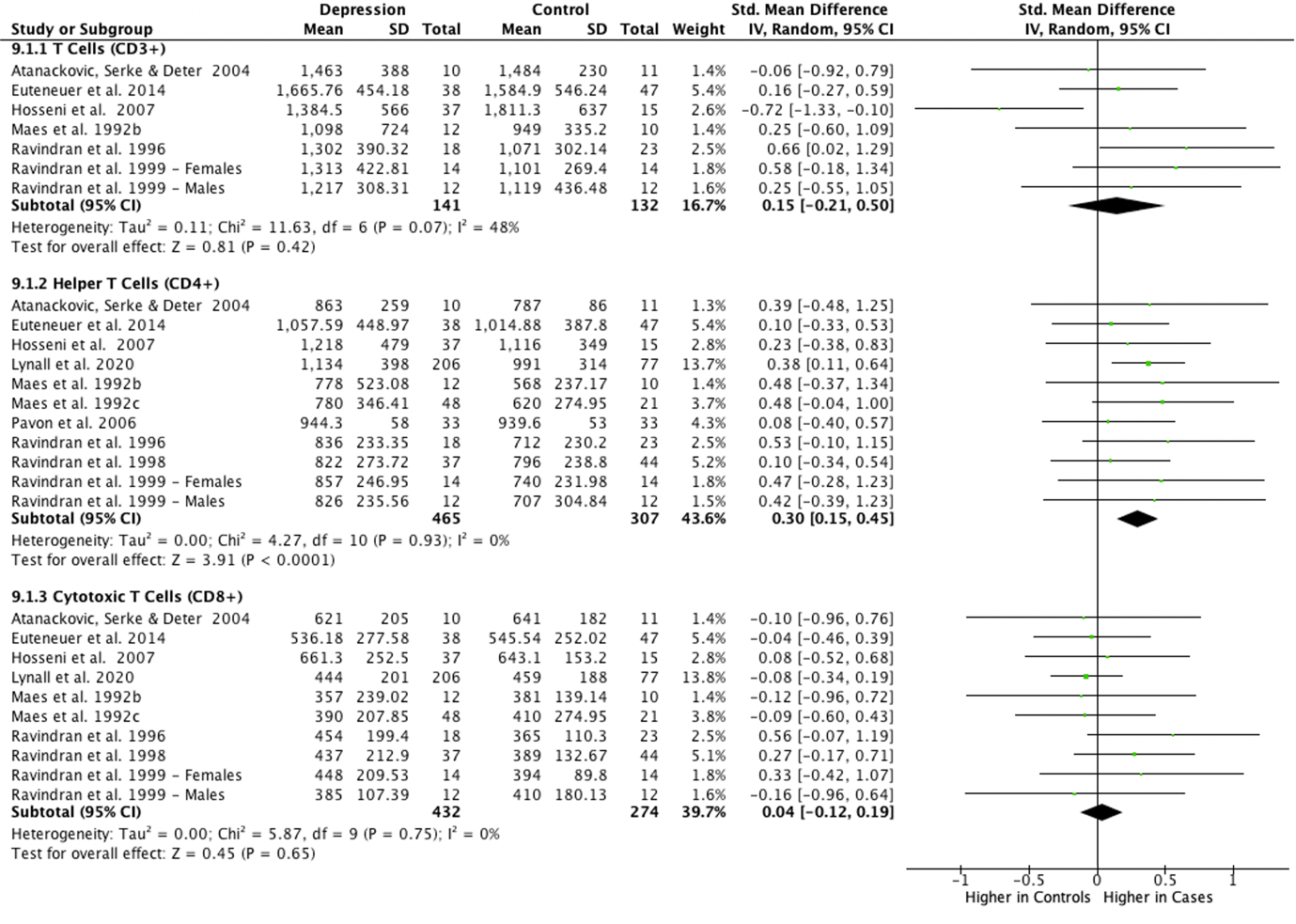
*

*Supplementary Figure 10*. Meta-analysis of relative percentage of T cell subsets in cases vs controls, including outliers for CD8^+^ T cells


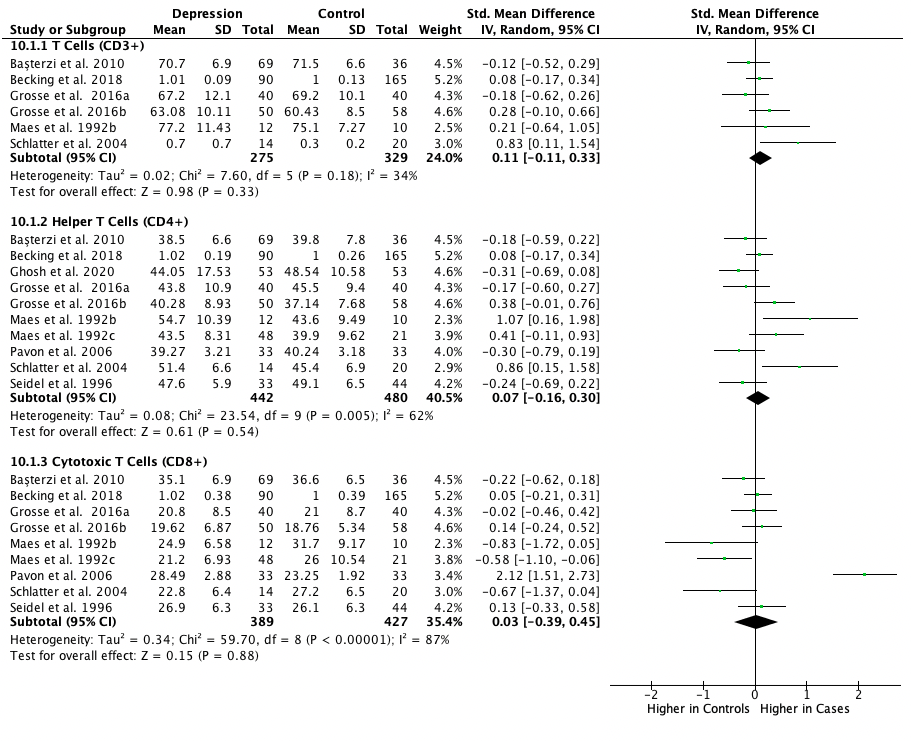


*Supplementary Figure 11*. Meta-analysis of relative percentage of T cell subsets in cases vs controls, excluding outliers for CD8^+^ T cells


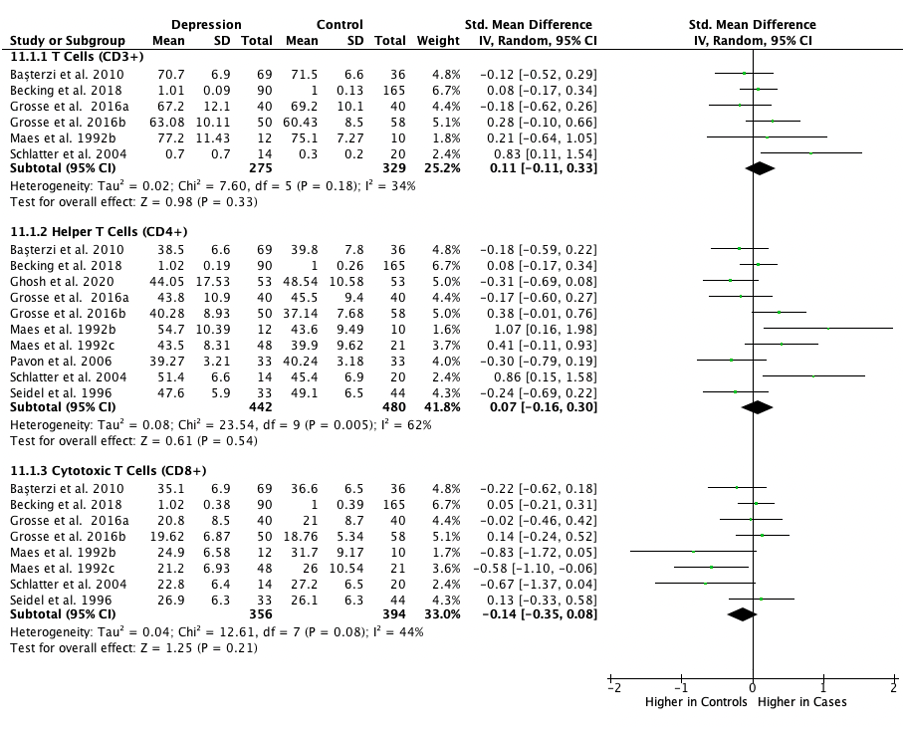


## *Supplementary Figure 12.* Meta-analysis of relative percentage of helper T cell subsets in cases vs controls


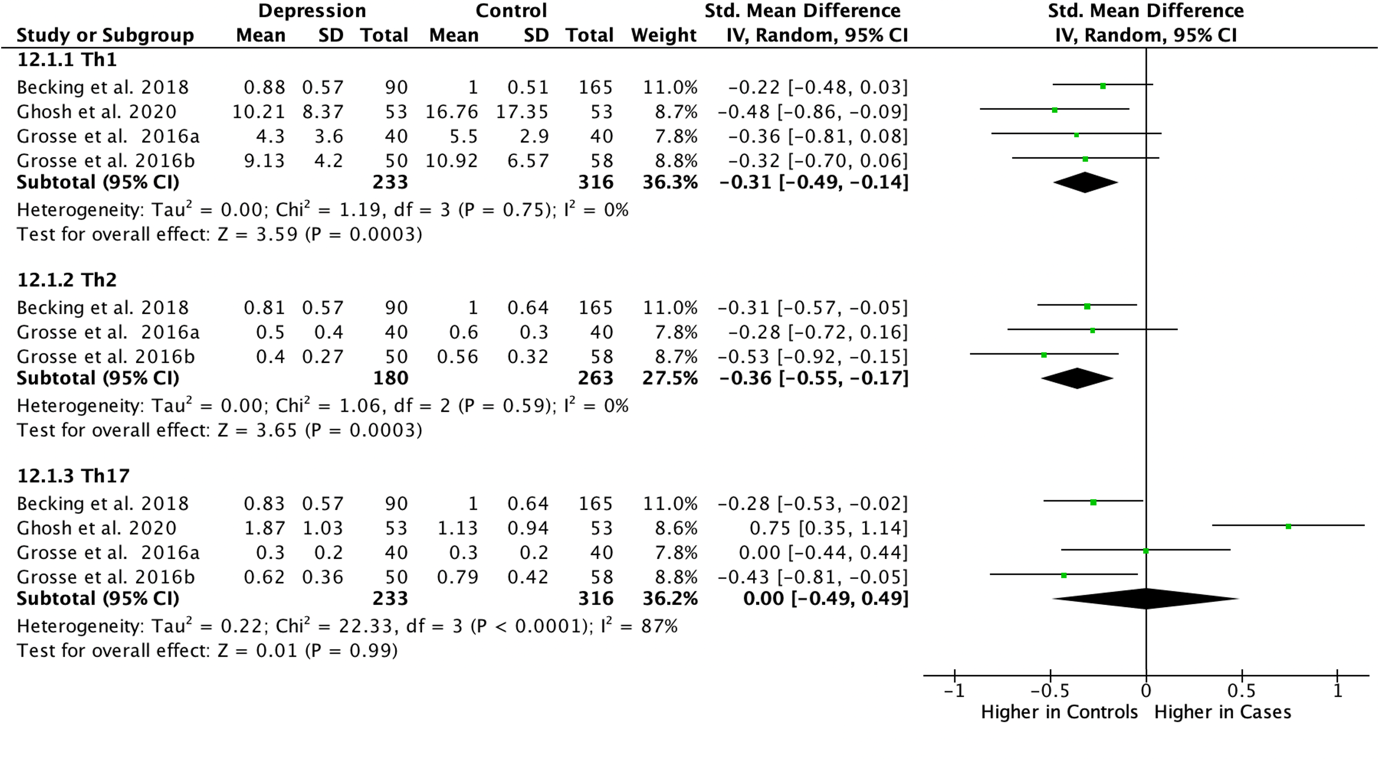


## *Supplementary Figure 13.* Meta-analysis of relative percentage of T regulatory cells in cases vs controls


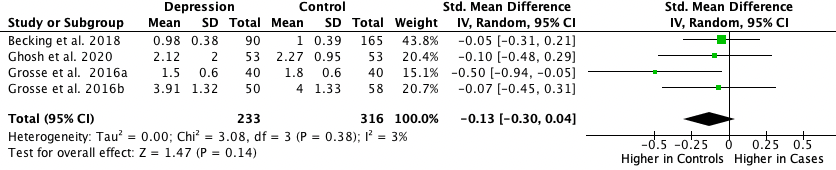


*Supplementary Figure 14.* Meta-analysis of mean absolute natural killer cell count in cases vs controls, excluding outliers


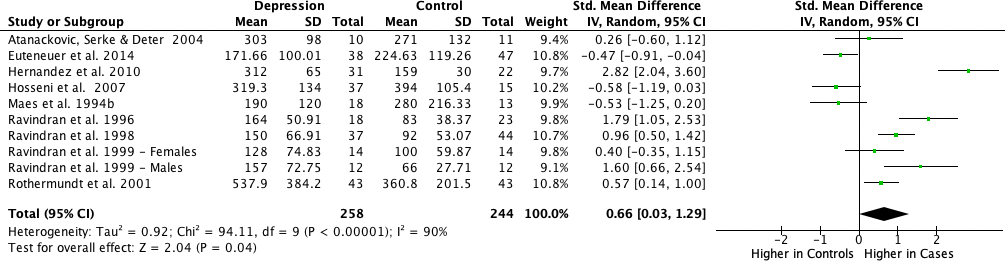


## *Supplementary Figure 15.* Meta-analysis of relative percentage of natural killer cells in cases vs controls, including outliers


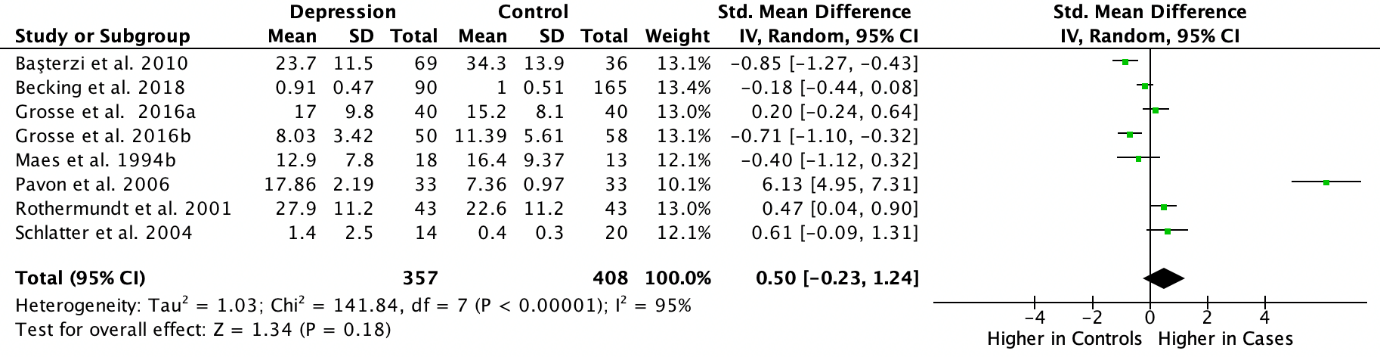


## *Supplementary Figure 16.* Meta-analysis of relative percentage of natural killer cells in cases vs controls, excluding outliers


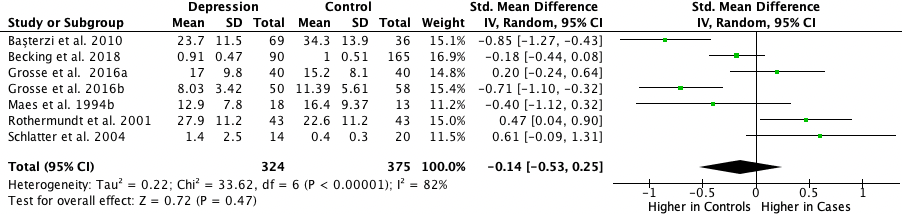


## *Supplementary Figure 17.* Meta-analysis of mean absolute B cell count in cases vs controls, including outliers


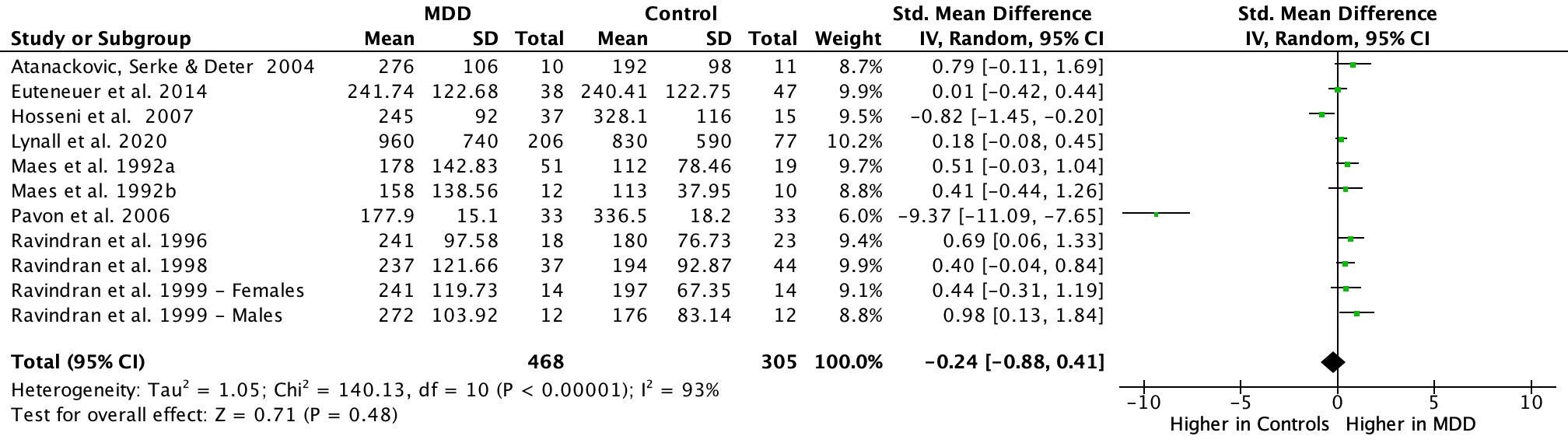


## *Supplementary Figure 18.* Meta-analysis of B cell relative percentage in cases vs controls, including outliers


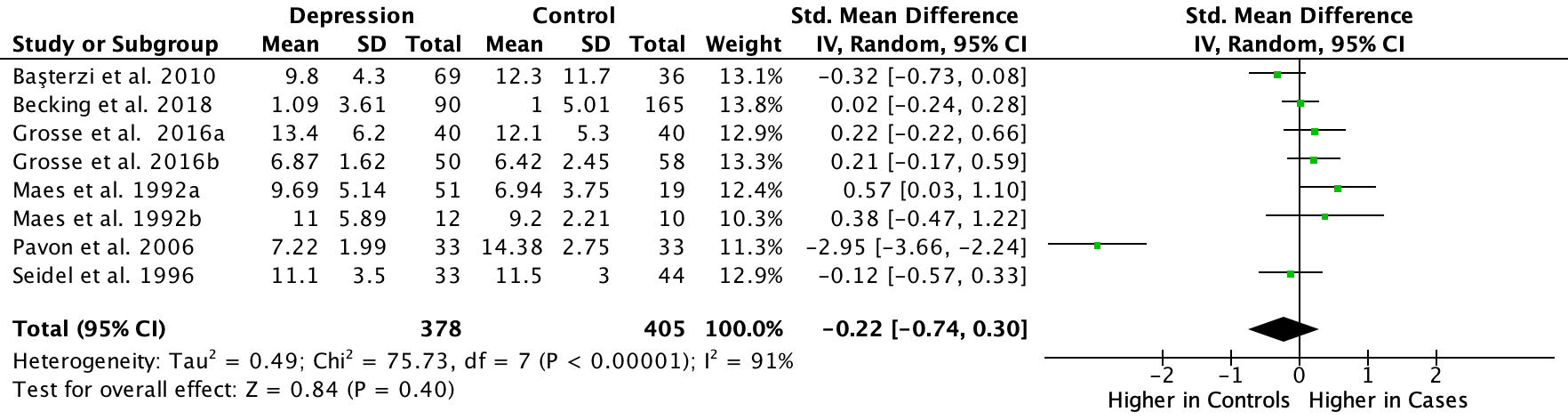


## *Supplementary Figure 19.* Meta-analysis of B cell relative percentage in cases vs controls, excluding outliers


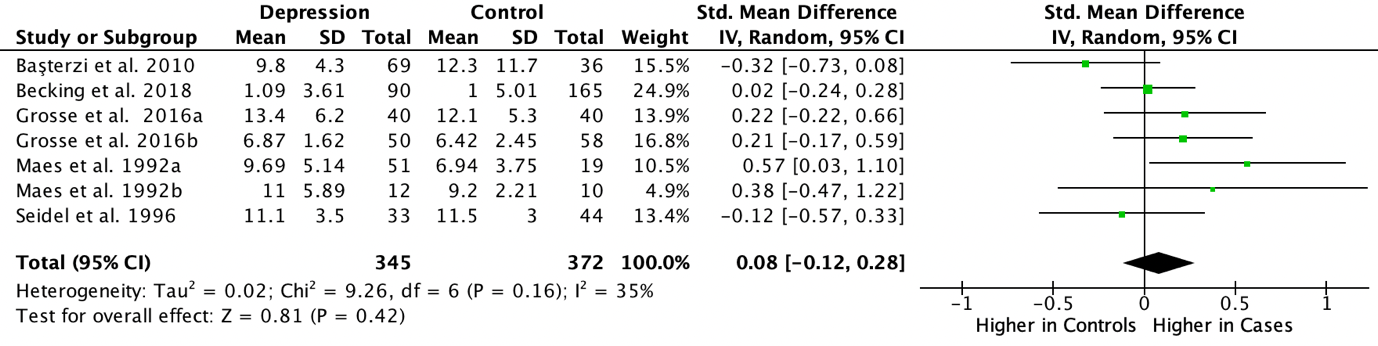


## *Supplementary Figure 20.* Meta-analysis of relative percentage of activated T cell subsets in cases vs controls

*
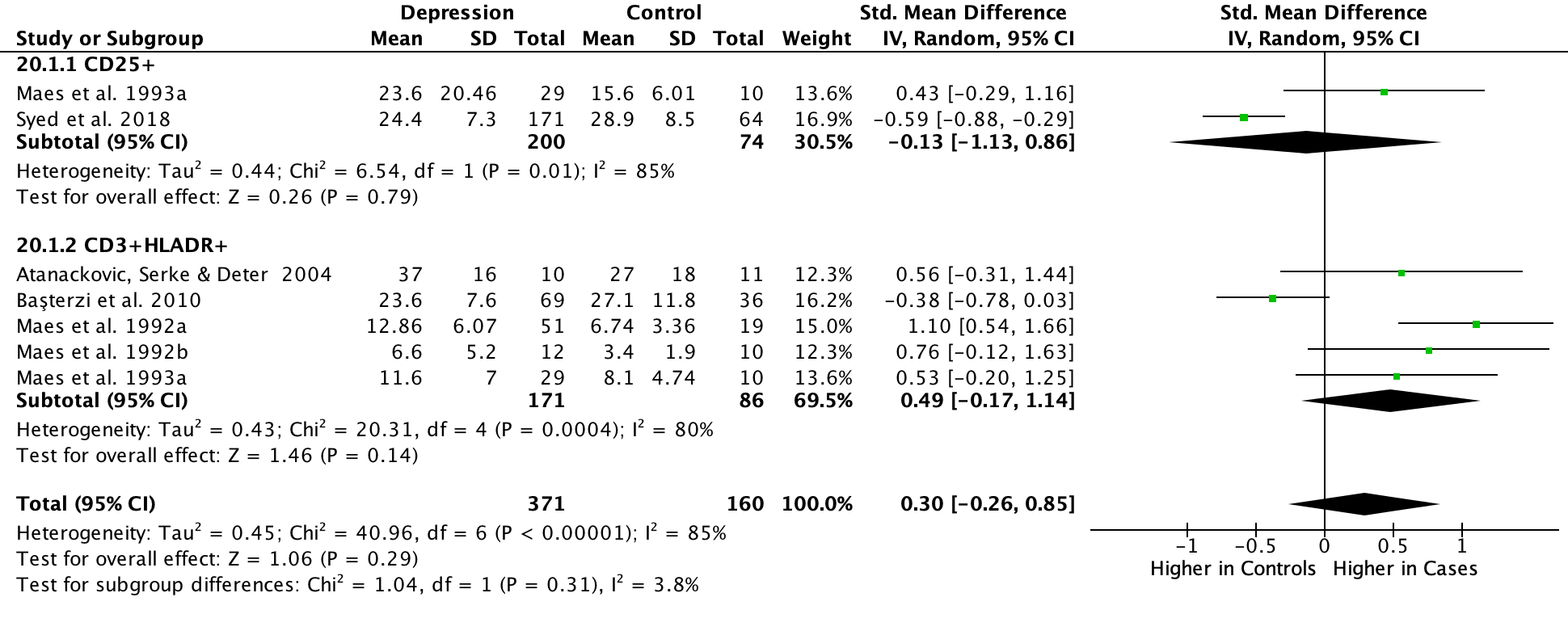
*

## *Supplementary Figure 21.* Meta-analysis of mean absolute CD4^+^/CD8^+^ ratio counts in cases vs controls, including outliers


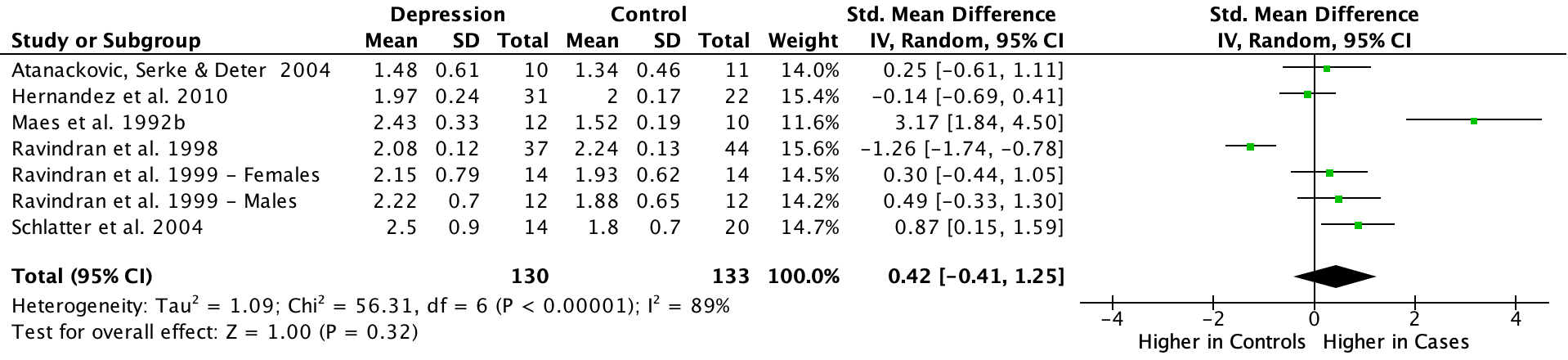


## *Supplementary Figure 22.* Meta-analysis of mean absolute CD4^+^/CD8^+^ ratio counts in cases vs controls, excluding outliers


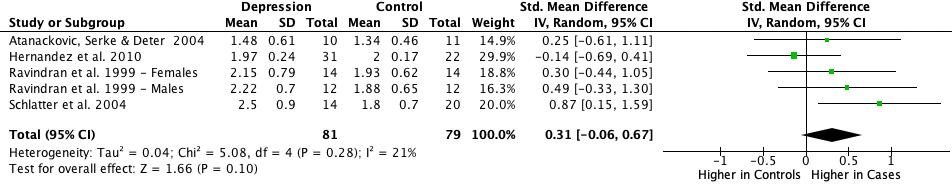


## *Supplementary Figure 23.* Meta-analysis of mean absolute naïve T cell counts in cases vs controls

*
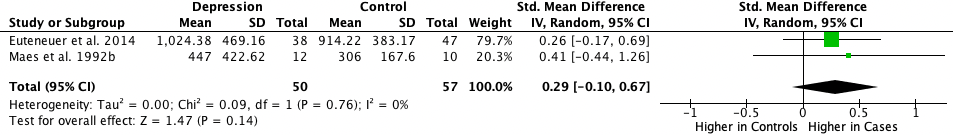
*

*Supplementary Figure 24.* Meta-analysis of relative percentage of naïve T cells in cases vs controls*
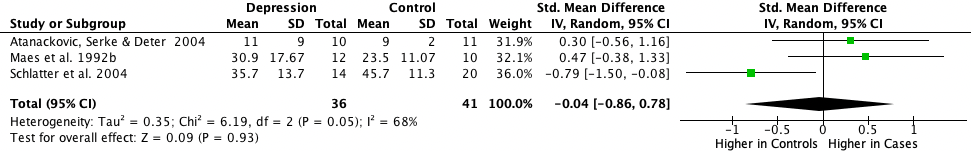
*

*Supplementary Figure 25.* Meta-analysis of relative percentage of memory T cells in cases vs controls

*
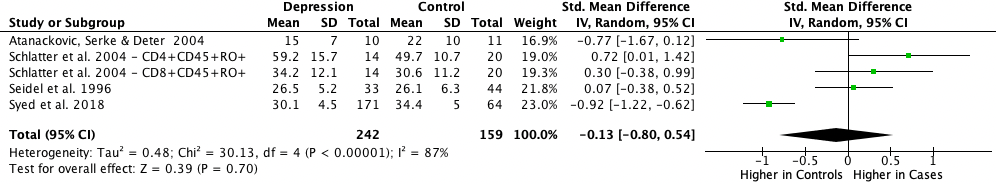
*
